# When our measurements are different every day: an ML-SEM simulation study on within-person nonuniform measurement bias in intensive longitudinal data

**DOI:** 10.3389/fpsyg.2026.1624037

**Published:** 2026-04-23

**Authors:** Georg Krammer

**Affiliations:** Institute of Business and Vocational Education, Johannes Kepler University Linz, Linz, Austria

**Keywords:** ecological momentary assessment, intensive longitudinal data, multilevel structural equation models, simulation, within-person nonuniform measurement bias

## Abstract

**Introduction:**

This simulation study evaluated how model fit in multilevel structural equation models (ML-SEM) is affected by within-person nonuniform measurement bias in intensive longitudinal data (ILD). This kind of bias would be given if item discrimination (i.e., their factor loadings) in multiple-item questionnaires varied within person across time. Prior simulation studies and ILD studies tend to assume no such within-person bias, while such a bias implies that relations within measurement points are not comparable across time.

**Methods:**

We simulating ILD under 450 conditions with various sample sizes, retesting frequencies, ICCs, and introduced within-person nonuniform measurement bias. We then investigated model (mis)fit in ML-SEM.

**Results:**

Type I error was well below nominal level. The χ^2^ statistic and CFI outperformed the other fit indices (RMSEA, SRMR-w, SRMR-b), with the effects being conditional on all design factors. Furthermore, even though standard software solutions readily provide SRMR specific for each level, results discourage their use due to poor and non-stable performance.

**Discussion:**

While ecological momentary assessment motivated our study, our findings are applicable to other research settings that yield data with the same generally hierarchical structure (e.g., ambulatory assessment, daily diary studies, experience sampling methods). We conclude with practical recommendations on samples sizes and re-testing frequencies to offer guidelines for evaluating ML-SEM in ILD.

## 1 Introduction

**Figure 1:**
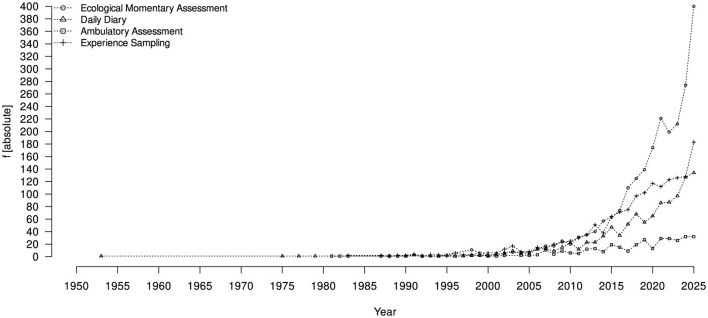
Results of a Open Alex search (done on March 17th 2026) for articles published between 1950 and 2025 containing either “ecological momentary assessment,” “daily diary,” “ambulatory assessment” or “experience sampling” in the title. Note that searching for either of these four terms in the title and additionally searching for “measurement” or “bias” resulted in 14 and 9 findings, respectively. For a full documentation of the underlying search including Open Alex API calls, please see Supplementary material uploaded in OSF (cf. Open Science Statement for the link).

There is currently a surge in the number of studies that use intensive longitudinal data, for instance, from *ecological momentary assessment* (EMA). Intensive longitudinal data has been around for quite a while in psychological research (e.g., Molenaar, 1985) and EMA has become widespread in many fields (Wrzus and Neubauer, 2023). A literature search for studies on intensive longitudinal data (EMA, but also “daily diary,” “ambulatory assessment,” or “experience sampling” in their titles) published between 1950

and 2025 shows a marked increase in recent years (see the summary of Open Alex searches in Figure 1). This increase is strongly stimulated by increasing accessibility of technological means of collecting such data (e.g., smartphone apps like ESMira: Lewetz and Stieger, 2024). Searching for publications that contain titles with either of these four keywords and additionally include “measurement” or “bias” resulted in merely 14 and 9 results, respectively.^1^ However, little attention is paid to issues of measurement, especially regarding within-person measurement bias in multiple-item questionnaires used for intensive longitudinal data.

To set the stage, we start with an example item taken from personality assessment that has already been used by others (see Grosz, 2024): “I am the life of a party” as an indicator of extraversion. It is highly likely that this item differs in how it functions between respondents who often go to parties and those who never attend them. We can address this difference from a measurement perspective by asking whether there is a measurement bias present and whether “I am the life of a party” functions differently for these two types of respondents. If it does, a between-person measurement bias is present which can, for example, be analyzed with multi-group structural equation models (Widaman and Reise, 1997). In intensive longitudinal data, we can translate this example to the within-person level and ask whether “I am the life of a party” functions differently at times when an individual respondent is attending many or few parties. If it does, a within-person measurement bias is present. For both the *between-* and the *within-person* case, party attendance may in itself be affected by respondents' extraversion. Psychometrically speaking, this would translate to a nonuniform measurement bias, which is indicated by factor loadings (λ) that differ depending on party attendance (Barendse et al., 2010; Penfield and Camilli, 2007).

We advise against assuming that no such bias is present *within person* when studying a latent trait such as extraversion in intensive longitudinal data. Instead, we recommend testing this assumption of no measurement bias, in particular, using multilevel structural equation modeling (ML-SEM: Lüdtke et al., 2007; Mehta and Neale, 2005; Muthén and Satorra, 1995; Stapleton, 2013) to complement exploratory approaches to addressing within-person measurement bias (e.g., Latent Markov Factor Analysis: Vogelsmeier et al., 2019). We make this recommendation because ML-SEM models have a long track record in evaluating psychological measures, do so in a confirmatory way, and are readily applicable to intensive longitudinal data. At the same time, little is known on how ML-SEM fare when within-person measurement bias is present. Note that we limited this simulation study to investigating nonuniform measurement bias, as (a) thoroughly addressing multiple biases for an emerging field is beyond the scope of one simulation study and (b) nonuniform measurement bias is the more extreme case among possible biases (Adolf et al., 2014) while having the practical implication that relations between constructs are not comparable if this type of bias is present (Widaman and Reise, 1997; Chen, 2008).

### 1.1 Within-person nonuniform measurement bias

We rely on the framework proposed by Adolf et al. (2014) to formalize and address nonuniform measurement bias within a person and how such a bias can be defined in intensive longitudinal data. To be able to evaluate the impact of within-person measurement bias by itself, we assume that there is no between-person measurement bias. Item response (y) of subject *i* at time point *t* is a linear function of intercepts (τ), factor loadings (λ), standing on latent variables (η) and residuals (ε). For one latent variable measured by *k* items,


$$\begin{array}{l} {\text{y}}_{\text{i},\text{t}}=\tau_{\text{i},\text{t}}+\lambda_{\text{i},\text{t}}*\eta_{\text{i},\text{t}}+\varepsilon_{\text{i},\text{t}}, \end{array}$$ (1)

where τ_i, t_ is a k × 1 vector of item intercepts, λ_i, t_ a vector of k × 1 factor loadings, η_i, t_ the standing on the latent trait, ε_i, t_ the measurement residuals, for which we assume a multivariate normal distribution with mean = 0 and covariance matrix Θ_i, t_. This yields y_i, t_ as a k × 1 vector of items responses. Note that Equation 1 can be extended to *q* latent variables by expanding η_i, t_ to a q × 1 vector of standings on the latent traits, and in turn by expanding the λ_i, t_ k × 1 vector to a k × q matrix, wherein each column consists of the factor loadings of the corresponding latent variable. Furthermore, Equation 1 allows variation of η_i, t_ with *i*, denoting interindividual differences between respondents, and with *t*, denoting for intensive longitudinal data that the standing on the latent trait of respondent *i* can change over time.

This simulation study was limited to investigating one latent variable and its measurement model. While there may be interindividual differences in η_i, t_, they are assumed to be time-invariant. This assumption is necessary to avoid confounding measurement bias with a change in the latent trait. We therefore arrive at


$$\begin{array}{l} {\text{y}}_{\text{i},\text{t}}=\tau_{\text{i},\text{t}}+\lambda_{\text{i},\text{t}}*\eta_{\text{i}}+\varepsilon_{\text{i},\text{t}}, \end{array}$$ (2)

where η_i_ is normally distributed, with the mean = 0 and a population variance of Φ_η_.

In Equation 2, intercepts τ and factor loadings λ may vary with respondents and time. The literature on measurement bias and measurement invariance focuses on the case where measurement bias occurs between subjects (Millsap, 2011; Widaman and Reise, 1997). This goes hand in hand with cross-sectional studies evaluating measurement invariance. Studies in which *t* does not vary yield


$$\begin{array}{l} {\text{y}}_{\text{i}}=\tau_{\text{i}}+\lambda_{\text{i}}^{*}\eta_{\text{i}}+\varepsilon_{\text{i}}, \end{array}$$ (3)

where all terms are defined as described above. In Equation 3, a nonuniform measurement bias would be present if λ_i_ varied systematically between distinct subgroups of respondents (Barendse et al., 2010; Penfield and Camilli, 2007).^2^ This constitutes a difference in item prioritization between distinct subgroups (cf. reprioritization: Oort, 2005; Sprangers and Schwartz, 1999). Affected items would not differentiate equally well across these distinct subgroups. If a nonuniform measurement bias is present, relations between constructs are not comparable (Widaman and Reise, 1997; Chen, 2008). This can be tested in multi-group structural equation models, and weak/metric measurement invariance would be established if no such nonuniform measurement bias were present.

While between-person nonuniform measurement bias is well-explored, the question arises whether measurement is invariant over time in intensive longitudinal data. Translating the notion of nonuniform measurement bias to this intensive longitudinal data context and our simulation study and using Equation (2) yields


$$\begin{array}{l} {\text{y}}_{\text{i},\text{t}}=\tau +\lambda_{\text{t}}^{*}\eta_{\text{i}}+\varepsilon , \end{array}$$ (4)

where we focus on nonuniform measurement bias. Accordingly, τ and ε are invariant between respondents and over time, while factor loadings may vary over time, which for intensive longitudinal data means that λ_t_ can vary *within person*. No measurement bias in λ_t_ would be present if the probability distribution of y_t_ depended only on η irrespective of the point in time *t* at which responses were collected. Thus, if


$$\begin{array}{l} \text{P}\left({\text{y}}_{\text{t}}|\eta ,\text{T}=\text{t}\right)=\text{P}\left({\text{y}}_{\text{t}}|\eta \right) \end{array}$$ (5)

is satisfied, no measurement bias is present. If, however, Equation 5 does not hold given the restrictions imposed in Equation 4, then λ_t_ would be affected by the measurement bias. We hereafter refer to this measurement bias that arises from systematic variation in λ_t_ dependent on time as *within-person nonuniform measurement bias*.

The literature refers to within-person nonuniform measurement bias using various terms in various contexts. Von Eye (2010) addressed the notion of measurement invariance precluding within-person measurement bias as *dimensional identity*. Sterba and Bauer (2010a,b) referred to its opposite as *interoccasion variability*. Along this line, Adolf et al. (2014) referred to *heterogeneity within individuals*. They all have in common the notion that a within-person uniform measurement bias is given if items of a multiple-item questionnaire are more relevant to a latent variable of interest on one measurement occasion than on another (Kim et al., 2023; Vogelsmeier et al., 2019).

Let us consider this with our initial example of “I am the life of a party” as an indicator of extraversion and party attendance as cause of within-person nonuniform measurement bias. A nonuniform measurement may be present if individuals fill in the extraversion item every day of the week, while not attending any parties during week days but doing so on weekends (similar to a “Thank God it's Friday” effect, cf. Camerman et al., 2024). In this example, the factor loadings λ_t_ of this extraversion item would differ between week days and weekends. While λ_t_ can vary *within person* at every measurement occasion, there is no conceptual limitation to how the underlying systematic effect may look. In the example above, λ_t_ would vary systematically as a function of the weekdays. Another systematic effect may look differently, for example, before and after major life events (Bleidorn et al., 2018) affecting party attendance resulting in λ_t_ before major life events to be different than λ_t_ after major life events. In common to these example effects on measurement is that they are systematically either given or not on a dichotomous basis (e.g., it is Friday or it is not Friday). However, on a more nuanced level, such effects may also enfold due to continuous environmental factors. A classical example would be stress, where a dichotomous stess/no-stress approach would be insufficient. To stay true to the extraversion example above, party attendance as factor may not only be dichotomous, but also continuous as in how many parties are being attended. As the current simulation study is the first of its kind, we focus on the dichotomous case, that is the influencing variable inducing the measurement bias affects a certain amount of measurement points in a dichotomous way (i.e., bias vs. no bias).

When considering effects of measurement bias, researchers should also consider the magnitude of this effect. Scholars have pointed to the fact that a bias that significantly affects model fit may still need to be evaluated for its practical significance (Meade, 2010). As with the logic of traditional null hypotheses significance testing, a dichotomous decision of the absence or presence of an effect does not paint the full picture; effect sizes are needed. Along this line, scholars have suggested measures of effect sizes to gauge how strong an effect of a measurement bias is (Meade, 2010). However, these suggestion stem from traditional analyses of differential item function or measurement invariance, that is they address the case where a third variable affects measurement of one latent variable in a single-level case. Given that no consensus on interpretation of such effect sizes measures has emerged and that furthermore, they are limited to single-level frameworks and not within-person measurement bias in intensive longitudinal data, such effect size measure are not directly applicable to this simulation study at hand. Before this background, we defined in this simulation study what a strong and what is a weak within-person nonuniform measurement bias is by considering what plausible factor loadings may be. Reviews of the literature on multilevel factor analyses indicate that factor loadings vary between λ = 0.41 and λ = 0.83 (Kim et al., 2016). Building on these findings, we define in this simulation study that a strong nonuniform measurement bias would constitute that a factor loading at the upper bound of this range was reduced to a factor loading falling outside of this bound (λ = 0.7 vs. λ = 0.2). At the same time, we defined a factor loading varying between the upper and the lower end of plausible factor loadings a low bias (λ = 0.7 vs. λ = 0.4).

Studies that evaluate how variables change and interact across time (e.g., Edershile and Wright, 2021) are faced with the caveat that the underlying assessment and consequently the conclusions drawn may be affected by measurement bias. This is particularly problematic because presence of a nonuniform measurement bias precludes that relationships to other constructs can be compared (Widaman and Reise, 1997; Chen, 2008) meaning in intensive longitudinal data, that relations at one time point to another variable cease to be comparable across other time points.

Unfortunately, it would seem that the absence of a within-person uniform measurement bias is more often assumed than tested. To the best of our knowledge, only Crawford et al. (2022) have recently, in a review article, tentatively addressed testing to confirm this absence. Methods have recently been proposed that are sufficiently flexible to recover parameters when within-person measurement bias in intensive longitudinal data is present (cf. cross-classified factor analysis: Kim et al., 2023), but they do not focus on evaluating multiple-item questionnaires. Furthermore, methods flexible enough to model such bias have also been proposed (cf. Latent Markov Factor Analysis: Vogelsmeier et al., 2019), but these rely on exploratory approaches not geared toward confirmatory testing of multiple-item questionnaires in intensive longitudinal data.

For the sake of completeness, we also need to distinguish within-person measurement bias from other biases that can arise in hierarchical and similar data structures. Consider measurement bias in large-scale assessments, such as cross-cultural studies, and longitudinal measurement invariance. For example, scholars have used multiple-item questionnaires to assess gender role attitudes across 59 countries (Lomazzi, 2018) and depression over time (Fried et al., 2016). In both cases, (i) the data could also be modeled as a hierarchical structure, with countries/time as nesting variable; and (ii) measurement bias is an issue, as scholars are eager to examine whether the construct of interest is equally meaningful and observable across countries/time. Methods such as alignment optimization (Pokropek et al., 2019) and longitudinal measurement invariance (Liu et al., 2017) are used to address measurement bias. While this bears a striking similarity to the measurement issues at the core of this simulation study, there is a fundamental difference. The examples above focused on interindividual differences and on whether these are reproduced across countries/time. In a multilevel framework, such questions would be tackled by addressing cluster bias (e.g., Jak et al., 2013), which is conceptually different from within-person measurement bias: Cluster bias addresses interindividual differences—that is, whether differences within one cluster replicate in other clusters—and is not concerned with differences within a cluster.

Furthermore, conceptual clarity of within-person nonuniform measurement bias also needs us to consider how person-specific such a phenomenon is and can be. To this end, we first need to clarify that obviously both within-person and between-person effects are constituted within the individuals we are studying (cf. Krammer et al., 2025). Put in other words, just because phenomena are studied based on variances derived from interindividual differences does not remove a phenomenon from being within a person; it states that we are studying it based on between-person differences. Turning to our within-person nonuniform measurement bias, we conceptualize it in this simulation study as a process that stems from individuals. At the same time, we follow that psychological science is heavily invested in discerning patterns that apply to multiple individuals, and applying these insights will very likely not fully explain the individual (Lamiell, 1998; Meehl, 1954). Accordingly, we see a within-person nonuniform measurement bias conceptualized at the individual level, but part of a pattern that also holds for other individuals. This is in line with stating that there are general laws discernible by psychological science, and that collecting more data on intraindividual differences may inform us on these (e.g., Bakan, 1955; Krammer, 2025; Renner et al., 2020).

### 1.2 Intensive longitudinal data and ML-SEM

Gathering intensive longitudinal data entails assessing participants several times. For example, scholars have assessed: mental health over 8 weeks with on average 158.66 measurement points per participant (Kellerman et al., 2022); smoking expectancies and lapse with on average 179.77 measurement points per participant (Potter et al., 2022); and drug use and flow-like states over 3 months with on average 153.90 measurement points per participant (Bertz et al., 2022). All these studies have in common that the data structure is multilevel: responses are nested within persons. Frequently, a two-level structure is considered for these data: Level 1 contains all responses of all participants across all measurement points. At level 2, these are nested within the respective participant. Another common nomenclature (e.g., in Mplus: Muthén and Satorra, 1995) uses *within* (i.e., within-person) for level 1 and *between* (i.e., between-person) for level 2.

EMA is not the only approach that yields intensive longitudinal data: Similar modes of assessment, such as ambulatory assessment (e.g., Crawford et al., 2022), daily diary studies (e.g., Rogoza et al., 2024) and experience sampling methods (e.g., Gabriel et al., 2019), also produce data with the same structure, and so does research in which respondents are nested within groups (e.g., Kim et al., 2016) or when a trait of one person is assessed by multiple respondents (e.g., students assessing their teachers: Krammer et al., 2019, 2021; Wagner et al., 2013). In all these cases, ML-SEM can be used to assess the psychometric soundness of the multiple-item questionnaires.

Intensive longitudinal data can also result from data collection from one subject. For example, researchers have used single-subject data of one respondent who answered questions related to alcohol consumption, mood, stress and health on 750 (!) consecutive days (von Eye et al., 2023, p. 21ff). While this also falls under intensive longitudinal data, we do not address single-subject research designs in this article, as these data do not require a multilevel extension of statistical analyses. Note, however, that several methods in the factor-analytic framework and their extensions to single/multiple subjects are applicable in this situation (e.g., dynamic SEM: DSEM; Asparouhov et al., 2018), which were introduced long ago (dynamic factor models: Molenaar, 1985). The interested reader is referred to the keynote article of Sterba and Bauer (2010a,b), its four comments (Ialongo, 2010; Molenaar, 2010; Mun et al., 2010; Von Eye, 2010) and the rebuttal (Sterba and Bauer, 2010b).

**Figure 2:**
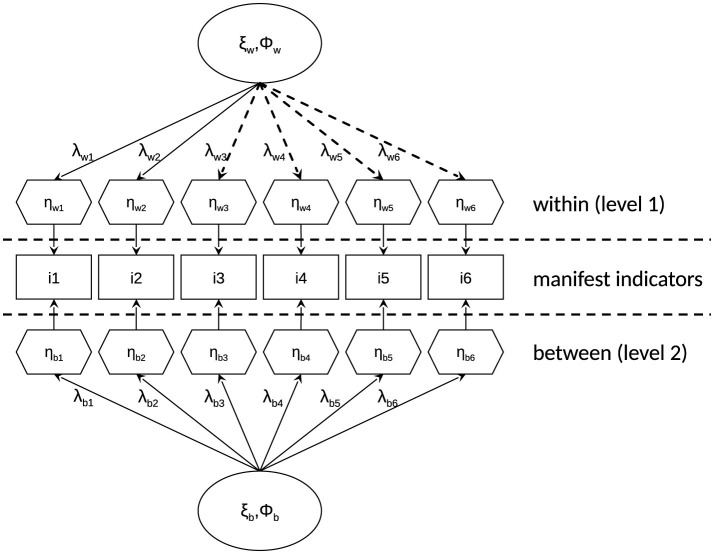
ML-SEM model with six indicator variables affected by one latent factor *within person* (level 1) and *between person* (level 2). At each level, latent variables have a true value ξ and variance Φ. The manifest indicators are parsed into their *between-person* (η_bi_; corresponding to the intercept τ in single-level SEM) and *within-person* (η_wi_; variance around η_bi_) components, both represented by hexagons. Each hexagon is accompanied by a residual variance ε. The ε values were omitted for better readability. The dashed lines represent the factor loadings for which we simulated the within-person nonuniform measurement bias.

For illustration, Figure 2 shows an ML-SEM model for a multiple-item questionnaire with six items *1–6*. While Equation 4 defines the latent variable η_i_ for the single-level case, ML-SEM assumes latent variables at each level—here, a measurement model at the *between* level (subscript b) and *within* level (subscript w). Accordingly, λ_t_ in Equation 4 is also given at the *between* and *within* levels, which yields λ_w1 − 6_ and λ_b1 − 6_ for the six items *1–6*.

Furthermore, ML-SEM defines τ in Equation 4 at the *between* level and models subjects' deviations from their *between* level intercept τ at the *within* level. At each level, the latent variables have their unique true values ξ_b_ and ξ_w_ and latent variances Φ_b_ and Φ_w_, respectively. Cross-sectional research traditionally examines the between-person latent variable, assuming that repeated measuring yields the true underlying values. It considers multiple subjects, the latent variable's variance Φ_b_ corresponds to interindividual differences. The within-person latent variable becomes accessible by assessing the same subjects multiple times, and its variance Φ_w_ corresponds to intraindividual differences. ML-SEM simultaneously models latent variables within-person and between-person. To do so, ML-SEM considers the variance/co-variance matrices at both levels and parses each variable observed (in multiple-item questionnaires: item *i*) into a between-person η_bi_ and a within-person η_wi_ component, each with their residual variances ε_bi_ and ε_wi_, respectively. Here, the between-person components η_bi_ are the average per participant, and the within-person components η_wi_ the deviations from the corresponding mean (i.e., group mean centered, with the participants being the nesting variable). Thus, indicators' intercepts τ_i_ as in single-level SEM exist only at the between-person level and correspond to the between-person component η_b_, while the deviations from these intercepts are represented by η_w_.

Note that the variance in η_b_ significance how pronounced the multilevel structure within intensive longitudinal data is. Should there be no such variance, there is no information to be gained knowing which participant is which participant. Accordingly, all data points would fulfill the assumption of being independent of each other, rendering single-level analyses viable. In turn, Equation 4 would not need to extended to a multilevel setting. Gauging how pronounced a multilevel structure within data is, is traditionally done by computing the ICC (Bliese, 2000). The ICC denotes how much variance can be explained in a variable by knowing its allocation to a higher level of data structure. This logic can be readily applied to intensive longitudinal data, where the ICC is given by the ration of the variances on the between level $\sigma_{between}^{2}$ and the variance of the within level $\sigma_{within}^{2}$:


$$\begin{array}{l} ICC=\frac{\sigma_{between}^{2}}{\sigma_{between}^{2}+\sigma_{within}^{2}} \end{array}$$ (6)

Each level can have its own set of latent variables, relations and covariates. To understand ML-SEM conceptually, it is important to note that structures and relations can be tested within each level and can be different within each level, while model constraints can also be imposed and tested across levels. Simulation studies have hitherto addressed measurement invariance across levels (e.g., Jak, 2019) or across clusters (e.g., Jak et al., 2013), whereas we sought to contribute by addressing measurement invariance within clusters (cf. Vogelsmeier et al., 2019).

Since a deep dive into the foundations of ML-SEM is beyond the scope of this article, we refer the interested reader to Muthén and Satorra (1995) for technical details, to the educational research of Lüdtke et al. (2007), as it traditionally deals with students nested in classes, and to the publications by Mehta and Neale (2005), Stapleton (2013), and Stapleton et al. (2016). For earlier research, see McArdle and Hamagami (1996), whose work emphasizes that, while implementation of ML-SEM in popular software packages such as Mplus and R's *lavaan* (Rosseel, 2012) has boosted its popularity, it has been possible for decades to coerce SEM software with the ability to handle multigroup SEM into handling ML-SEM.

What do previous simulation studies tell us about ML-SEM? Scholars have analyzed (1) factorial invariance across the *within* and *between* levels and related estimation problems (Jak, 2019), (2) collinearity at the *within* and *between* levels and bias in the parameter estimates (Can et al., 2015), (3) measurement invariance assumptions across clusters by introducing uniform and nonuniform measurement bias at the *between* level (Jak et al., 2013) and (4) evaluating latent variables' group mean differences in an ML-SEM framework (Kim and Cao, 2015). Simulation studies have also evaluated model fit in ML-SEM (Hsu et al., 2015), assuming no misspecifications within clusters. Hsu et al. found the χ^2^ statistic to have a slightly higher Type I error rate (8.358%), while according to Hu and Bentler (1999), the cut-offs of fit indices had a minimal Type I error (SRMR at the between level had the highest Type I error: 0.012%). The same fit indices performed better in detecting complex rather than simple model misspecification, and—except for the SRMR at the *between* level (SRMR-b)—they were not sensitive to *between*-level model misspecification. Accordingly, scholars have emphasized (Kim et al., 2016) that detecting model misfit in ML-SEM is driven mainly by level 1 misfit. These findings imply that for intensive longitudinal data ML-SEM is sensitive to model misfit at the within-person level. We simulated various between-person differences (D_b_ as means of introducing variance on η_b_ to emphasize the multilevel structure) thereby driving intraclass correlation (ICC). This was based on Equation 6. Increasing the between-person differences increases $\sigma_{between}^{2}$ in Equation 6, thereby increasing the ICC.

All the simulation studies mentioned above assume that for all measurement occasions within level 1 clusters (be it across nested respondents or across time points of measurement, etc.) the measurement models are not affected by measurement bias at the within-person level. In other words, the misspecifications modeled are not misspecified at the within-person level.

### 1.3 ML-SEM: advantages and disadvantages

Reviews are starting to emerge that call attention to the severe lack of studies that report psychometric properties of multiple-item questionnaires in intensive longitudinal data (e.g., for ambulatory assessment: Trull and Ebner-Priemer, 2020).^3^ ML-SEM (Lüdtke et al., 2007; Mehta and Neale, 2005; Muthén and Satorra, 1995; Stapleton, 2013) offers a promising way of examining the psychometric properties of multiple-item questionnaires in intensive longitudinal data and of testing dimensional identity. A recent example of its application can be found in personality research, where ML-SEM models have been employed to scrutinize if and how well-established multiple-item questionnaires can be used successfully with intensive longitudinal data (Grosz, 2024; Rogoza et al., 2024). ML-SEM thus adds value by providing a confirmatory test of psychometric instruments.

ML-SEM also complements other methods: It can be used to evaluate psychological measures before continuing with other analyses, such as dynamic SEM (DSEM: Asparouhov et al., 2018). ML-SEM models can also be employed to complement exploratory approaches such as Latent Markov Factor Analysis (Vogelsmeier et al., 2019), because they are confirmatory in nature and thus allow assessment of model fit as opposed to contrasting different parameter estimations obtained from exploratory models (e.g., via BIC or og-likelihood-based selection criteria: Schmitt et al., 2024; Vogelsmeier et al., 2023). The long-standing tradition in the SEM literature of evaluating model fit also makes ML-SEM a complementary procedure that is geared toward parameter estimation in intensive longitudinal data, such as those in cross-classified factor analysis (cf. Kim et al., 2023). Taken together, ML-SEM allows researchers and practitioners not only to address measurement bias, but also to evaluate multiple-item questionnaires in a confirmatory fashion.

Applying ML-SEM generally—and thus also in the context of intensive longitudinal data—brings with it the issue of how to evaluate model fit. Existing and commonly cited guidelines for evaluating model fit in SEM (e.g., Byrne, 2013; Hu and Bentler, 1999; Marsh et al., 2004; Schermelleh-Engel et al., 2003) were developed for single-level SEM (Kim et al., 2016), and computing fit indices in ML-SEM is already problematic because basic questions such as “What is the sample size?” are up for debate (Mehta and Neale, 2005). Accordingly, scholars investigating intensive longitudinal data with ML-SEM report the fit indices they would normally report (e.g., CFI, RMSEA, SRMR: Kim et al., 2016), with some refraining from commenting on this limitation (e.g., Lüdtke et al., 2007) and others repeatedly commenting on it while not modifying their practice (cf. Krammer et al., 2019 vs. several years later Rogoza et al., 2024).

Little is known about how sensitive ML-SEM models are to measurement bias in intensive longitudinal data and what fit criteria are appropriate (guidelines for evaluating model fit are based predominantly on the single-level SEM: Kim et al., 2016). It seems that, as with studies using intensive longitudinal data, simulation studies and reviews of ML-SEM also assume the absence of measurement bias at the lowest level (e.g., Can et al., 2015; Hsu et al., 2015; Jak, 2019; Jak et al., 2013; Kim et al., 2016; Kim and Cao, 2015). Against this background, this paper presents a simulation study to assess (1) the Type I error of ML-SEM applied to intensive longitudinal data and (2) the power of ML-SEM to detect a violation of dimensional identity as caused by within-person nonuniform measurement bias.

### 1.4 The current simulation study

In summary, researchers are collecting increasing amounts of intensive longitudinal data, but they rarely test underlying assumptions, such as the absence of within-person measurement bias; even the psychometric properties of multiple-item questionnaires are under-reported (Trull and Ebner-Priemer, 2020). Since ML-SEM offers a compelling way of evaluating psychological measures in intensive longitudinal data (Rogoza et al., 2024), we set out in this simulation study to examine how they fare when confronted with within-person nonuniform measurement bias.

### 1.5 Simulation design

In accordance with prior ML-SEM simulation studies (Hsu et al., 2015; Jak, 2019; Kim and Cao, 2015), we examined the effects on Type I error and power of a range of data sizes by varying the number of level 2 clusters assessed with various numbers of data points per cluster in intensive longitudinal data: this corresponds to varying the number of participants (*n*) and the number of times (*t*) they were re-tested, respectively. As extending single-level analyses to multiple levels is necessary only when the hierarchical data structure is sufficiently pronounced (Hox et al., 2017), we simulated various between-person differences (D_b_ as means of introducing variance on η_b_ to emphasize the multilevel structure; cf. $\sigma_{between}^{2}$ in Equation 6) thereby driving intraclass correlation (ICC). If there was no unique variance component to the participants, within-person nesting would not be necessary. In such a scenario, which would be indicated by negligible ICCs, a single-level analysis would suffice. Accordingly, and like prior ML-SEM simulation studies (Hsu et al., 2015; Jak, 2019; Kim and Cao, 2015), we varied ICCs to vary how pronounced the hierarchical data structure was.

We limited this simulation study to examining within-person nonuniform measurement bias in order not to overburden it by addressing multiple biases in an emerging field. Furthermore, nonuniform measurement bias is the extreme case (Adolf et al., 2014) and therefore warrants more pressing attention. A within-person nonuniform measurement bias in ML-SEM for intensive longitudinal data implies that the factor loadings within a person (λ_wi_) vary across time. To the best of our knowledge, no prior simulation study has assessed the power of ML-SEM to detect within-person nonuniform measurement bias. On the contrary, prior simulation studies that tested ML-SEM have assumed that measurement is equivalent within a cluster (e.g., Hsu et al., 2015; Jak, 2019; Jak et al., 2013).

In this simulation study, we examined χ^2^ statistic, CFI, RMSEA, SRMR-b and SRMR-w. We chose this subset of fit-indices as they are heavily used fit indices that are popular in the SEM literature (Byrne, 2013; Hu and Bentler, 1999; Marsh et al., 2004; Schermelleh-Engel et al., 2003). Recent state-of-the-art simulation studies continue to rely on this subset of fit indices, which attests to their relevance to SEM (Heene et al., 2024). With the exception of TLI, these are the fit indices that the most extensive ML-SEM simulation study to date examined (Hsu et al., 2015). While the SRMR is not without critique (Marsh et al., 2004), it is a fit index readily available for each level within ML-SEM, and thus SRMR-b and SRMR-w should be included. Lastly, the high correlations between fit indices indicate that multiple fit indices may not provide unique information above and beyond each other (Kim et al., 2016), which supports our decision to use five dominant fit indices.

#### 1.5.1 Hypotheses

Due to this simulation study being the first of its kind, our hypotheses were guided by two things. First, we were guided by the most extensive ML-SEM simulation study to date (cf. Hsu et al., 2015). Note that while Hsu et al. did not aim specifically to contribute to handling intensive longitudinal data in their simulation study, they did so implicitly because intensive longitudinal data is multilevel in nature, with participants being the nesting structure. Second, we were guided by the following line of reasoning: A within-person nonuniform measurement bias is not a model misspecification at the between-person level but at the within-person level. It follows that approaches that are conceptualized at the between-person level (i.e., SRMR-b) are likely outperformed by their counterparts at the within-person level (i.e., SRMR-w), while common methods of evaluating fit (χ^2^, CFI and RMSEA) may retain at least some of their power (for a similar line of reasoning see Kim et al., 2016).

Our first set of hypotheses is related to Type I error rates and is based on prior findings. We hypothesized that the χ^2^ statistic would show a nominal Type I error rate. Hsu et al. (2015) found elevated Type I error rates, but simulated additional misfit explaining these rates). For the fit indices, we expected poorer performance of SRMR-b as compared to CFI, RMSEA and SRMR-w (Hsu et al., 2015).

Our second set of hypotheses is related to detecting induced misfit. First, we hypothesized that the χ^2^ statistic has adequate power given sufficiently large data sets. This is in line with previous simulation studies (Hsu et al., 2015) and scholars who emphasized the importance of the χ^2^ statistic (Greiff and Heene, 2017). As for the Type I error rates, we hypothesized that CFI, RMSEA and SRMR-w would outperform SRMR-b. We expected increasing power with more participants (*n*) and participants being tested more often (*t*). Furthermore, we also expected an effect of how pronounced the hierarchical data structure was (i.e., varying between-person differences *D*_b_ resulting in varying ICCs). This was based on the notion that within intensive longitudinal data differences between persons are necessary to denote a hierarchical data structure. Accordingly, data with higher between-person differences yield higher ICCs (cf. Kim and Cao, 2015, but also Equation 6). As the measurement bias in focus of this simulation study stems from the within-person level, a more strongly pronounced within-person level lends more weight to misspecifications at this level. As no prior studies analyzed these effects and no prior literature linked measurement bias on the within-person level to how pronounced multilevel structures in intensive longitudinal data are, we postulated this as an exploratory hypothesis. Finally, we hypothesized that the more items were affected by a nonuniform measurement bias and the stronger this bias was, the higher the power would be.

## 2 Method

### 2.1 Simulation design

Data were simulated under 3 × 5 × 3 × 5 × 2 = 450 conditions with 1,000 data sets each. First, we varied the amount of data collected by varying sample size and how often measurement was repeated. To this end, we simulated three sample sizes with n ∈ {50, 100, 200} and five numbers of testing per subject with t ∈ {10, 20, 30, 50, 80}. The sample sizes were chosen to accommodate common sample sizes in intensive longitudinal data and the number of testing was chosen in accordance with EMA reviews (cf. Wrzus and Neubauer, 2023). We then varied how pronounced the hierarchical data structure was by varying the between-person differences *D*_*b*_ on the latent trait ξ_b_ (cf. Kim and Cao, 2015). We thus simulated ξ_b_ drawn from a normal distribution centered around 0 and with *SD*_*b*_ ∈ {1, 2, 3}. We then simulated five qualitatively different types of nonuniform measurement bias *b* ∈ {none, 1, 2, 3, 4}: no bias to assess Type I error (termed *none*) and four different nonuniform measurement bias conditions (termed *1, 2, 3*, and *4;* see Table 1 and for a graphical representation the dashed lines in Figure 2) to assess power. Finally, we used two strengths of nonuniform measurement bias (low, high). We realized this by varying the differences between factor loadings with Δλ_wi_ ∈ {0.3, 0.5}.

**Table 1:** Overview of the within-person factor loadings (λ_wi_) manipulated for the five nonuniform bias conditions across the six items (cf. Figure 2).

Bias condition	Practical interpretation	λ_w1_	λ_w2_	λ_w3_	λ_w4_	λ_w5_	λ_w6_
None	No bias	=	=	=	=	=	=
1	Small bias, one item affected, consider removing item	=	=	=	=	=	0.7 vs. 0.2/0.4
2	Moderate bias, two items affected, scale needs revising	=	=	=	=	0.7 vs. 0.2/0.04	0.7 vs. 0.2/0.4
3	Moderate bias, two items affected, scale needs revising	=	=	0.7 vs. 0.2/0.4	=	0.2/0.4 vs. 0.7	=
4	Severe bias, four items affected, scale compromised	=	=	0.7 vs. 0.2/0.4	0.7 vs. 0.2/0.4	0.2/0.4 vs. 0.7	0.2/0.4 vs. 0.7

Equal signs denote that λ_wi_ was equal (i.e., always 0.7) across all measurement points of the subject. Otherwise, λ_wi_ of the 1st half of measurement points vs. the 2nd half is given (0.4 for the low-strength condition and 0.2 for the high-strength condition).

### 2.2 Simulation procedure

The simulation and analyses were carried out in R (R Core Team, 2023b), with the packages *lavaan* (Rosseel, 2012), *psych* (Revelle, 2019), *multilevel* (Bliese, 2022), *parallel* (R Core Team, 2023a) and *doParallel* (Corporation and Weston, 2022). We simulated 1,000 data sets per condition. The ML-SEM model used for all simulation conditions is shown in Figure 2: We assumed a model with six items, as this matches a typical number of item in multiple-item questionnaires in research using intensive longitudinal data (e.g., Study 1 and Study 3b in Rogoza et al., 2024) and in previous ML-SEM simulation studies (e.g., Hsu et al., 2015). We drew the between-person latent trait ξ_b_ from a random normal distribution and let it vary around μ = 0 with *SD*_*b*_ ∈ {1, 2, 3} to introduce low, medium and high ICCs, respectively. To simulate more realistic item responses, we drew for each subject a skewness and a kurtosis value in the range −0.5 to +0.5 from a uniform distribution for item-response simulation. We set the latent and manifest variances to unity to force standardized factor loadings, which we set *within person* to λ_wi_ = 0.7. This value was chosen because (1) it aligns with what has been reported for published ML-SEM models (Kim et al., 2016) found level 1 factor loadings with an average range of 0.41–0.83) and prior simulation studies (Hsu et al., 2015; Kim and Cao, 2015), (2) it is a realistic value for multiple-item questionnaires in intensive longitudinal data (cf. Study 1 and Study 3b in Rogoza et al., 2024) and (3) it leaves ample scope for variation across time points of measurement to introduce within-person nonuniform measurement bias. This yields factor loadings at the upper and lower range of what previous reviews have reported (Kim et al., 2016: λ between 0.41 and 0.83), as well as one conditions with factor loadings clearly falling outside of this range.

The nonuniform measurement bias was introduced by varying the factor loading λ_wi_ between the 1st and 2nd half of a subject's re-testing. For example, in a condition where each subject was tested 30 times, for the first 15 re-testings Item 6 had λ_w6_ = 0.7, while for the last 15 re-testings it had λ_w6_ = 0.4 in the *low-strength* condition and λ_w6_ = 0.2 in the *high-strength* condition. This simplification was chosen as in ML-SEM there is no information contained in which measurement point is which measurement point, above and beyond which were the same and which were different measurement points. Accordingly, it would be an equivalent simulation study to introduce the nonuniform measurement for the factor loadings λ_wi_ of every second measurement point. First, we simulated no measurement bias (bias condition none). Then, the four different types of nonuniform measurement bias were simulated by varying the factor loadings as summarized in Table 1 and graphically indicated by the dashed lines in Figure 2. One item was affected in bias condition 1, two items in bias conditions 2 and 3, and four items in bias condition 4. Bias conditions 2 and 3 were variations of each other. In bias condition 2, the nonuniform measurement bias affected both items in the same way (2nd half of measurement points had a reduced within-person factor loading); in bias condition 3, this was varied between the two items, meaning one item was affected as in condition 2, and for the other one the nonuniform measurement bias was flipped (1st, not 2nd, half of measurement points had a reduced within-person factor loading). Lastly, bias condition 4 extended bias condition 3, with a total of four items being affected by measurement bias. Across all conditions, items 1 and 2 were always measurement invariant; that is, λ_w1_ and λ_w2_ were not affected by a nonuniform measurement bias and thus equal across the 1st and 2nd halves of measurement points.

Data were simulated following the person-oriented focus of intensive longitudinal data by simulating each person individually and then combining the data to one data set. That is, while the simulation produced data per individual, the person-related parameters were drawn from overarching distributions. Assuming *t* measurement points, the *within* level of Figure 2 was thus simulated *t*/2 times with one set of factor loadings and t/2 times with another set of factor loadings (cf. Table 1 and the dashed lines in Figure 2), which were combined to form the data of each subject simulated. This procedure was repeated *n* times and combined to one dataset. To achieve more realistic data, the item responses thus simulated for the six items were transformed into a visual analog scale (range: 0–100). A visual analog scale was chosen because it is a common and promising response format for intensive longitudinal data (e.g., Jauk et al., 2023a,b; Maliske et al., 2023).

### 2.3 Analyses per data set

After simulating a data set, we computed ICCs per item and fitted the ML-SEM model shown in Figure 2 to the data. The model was identified by setting the first factor loading to unity. All ML-SEM models were estimated with a maximum likelihood estimator, as item responses were given on visual analog scales. Since the data were well-distributed (all skewness and kurtosis values | < 0.5|) and no data were missing, the standard maximum likelihood estimator was used (no robust standard errors, etc.). In doing so, no convergence issues were encountered.

Across the 1,000 data sets per condition, we first recorded the mean ICCs per item. We then evaluated how often the χ^2^ statistic was significant (*p* < 0.05) and computed the mean fit indices and their standard deviations (cf. Hsu et al., 2015). As the literature offers various suggestion for CFI, RMSEA and SRMR cut-offs (e.g., Byrne, 2013; Hu and Bentler, 1999; Marsh et al., 2004; Schermelleh-Engel et al., 2003), we used several and documented for each how often model fit was evaluated as positive. The cut-offs employed were 0.99, 0.95, and 0.90 for CFI, 0.10, 0.08, and 0.06 for RMSEA and 0.08 and 0.11 for SRMR-w and SRMR-b. When evaluating the power, we assumed good performance given a power of 80%.

## 3 Results

### 3.1 Hierarchical data structure

First, we examined the ICCs across all conditions (see Table A1 in Appendix). Across all conditions and items, assuming *SD*_b_ = 1 yielded an average ICC of 0.219 (*SD* = 0.044), *SD*_b_ = 2 an average ICC of 0.515 (*SD* = 0.082) and *SD*_b_ = 3 an average ICC of 0.693 (*SD* = 0.089). We therefore concluded that we had successfully manipulated the prominence of the hierarchical data structure of the simulated data. Looking more closely at the ICCs also revealed that if λ_wi_ was equal across items, the respective items' ICCs were also equal. However, items affected by within-person nonuniform measurement bias exhibited consistently lower ICCs than unaffected items. Recalling how different *SD*_b_ values yielded different ICCs, we hereafter refer to the three different *SD*_b_ conditions as low, medium and high ICC conditions.

### 3.2 Type I error

Next, we investigated the Type I error by examining model fits of *bias condition none*. We report all average model fit indices and the rates of fulfillment of the fit-index cut-off criteria (employing commonly used SEM cut-off values) in Tables A2, A3 in Appendix, respectively. Across all conditions, the χ^2^ statistic had a Type I error between 0% and 0.5%, and even with the strictest cut-offs the fit indices had at most a Type I error of 0.6%. Accordingly, across all conditions average CFIs were at 1.000, average RMSEAs between 0.000 and 0.001, average SRMR-b between 0.000 and 0.017, and average SRMR-w between 0.003 and 0.020. In summary, we found the Type I error to be well below the nominal level. Taken together, the χ^2^ statistic, CFI, RMSEA, SRMR-b and SRMR-w performed very well when no within-person nonuniform measurement bias was present.

### 3.3 Power: one biased item

For *bias condition 1*, none of the approaches were sufficiently powerful, neither the χ^2^ statistic (rejecting at best 0.4%) nor the fit indices (rejecting at best 0.5% even with the strictest cut-offs). We report all average model fit indices in Table A4 in Appendix, and the percentage of how often fit-index fulfilled their cut-offs in Table A5 in Appendix. Taken together, neither the χ^2^ statistic nor any of the fit indices (CFI, RMSEA, SRMR-b and SRMR-w) identified misfit when the bias was present.

### 3.4 Power: two biased items

We then examined the two conditions where within-person measurement bias affected two items (*bias conditions 2* and *3*). We report the percentage of how often fit-index fulfilled their cut-offs in Table 2. For all average model fit indices, the interested reader is referred to Table A6 in Appendix. These bias conditions had in common that neither SRMR-b (at best it rejected 0.2%) nor SRMR-w (at best it rejected 26.1%) exhibited sufficient power.

**Table 2:** Percentages of fit indices above/below their cut-off according to common SEM cut-offs for various ICCs (low, med. = medium, high), number of participants (*n*), number of re-tests (*t*) and for the two nonuniform bias conditions where two items were affected (Bias) with low and high bias.

				Low											High										
				CFI				RMSEA			SRMR-b		SRMR-w		CFI				RMSEA			SRMR-b		SRMR-w	
Bias	ICC	*n*	*t*	*p*<0.05	≥0.99	≥0.95	≥0.90	<0.06	<0.08	<0.10	<0.08	<0.11	<0.08	<0.11	*p*<0.05	≥0.99	≥0.95	≥0.90	<0.06	<0.08	<0.10	<0.08	<0.11	<0.08	<0.11
2	Low	50	10	2	0.993	1	1	1	1	1	1	1	1	1	21	0.930	1	1	1	1	1	0.997	1	1	1
			20	8	0.995	1	1	1	1	1	1	1	1	1	120	0.896	1	1	1	1	1	1	1	1	1
			30	7	1	1	1	1	1	1	1	1	1	1	233	0.877	1	1	1	1	1	1	1	1	1
			50	18	1	1	1	1	1	1	1	1	1	1	572	0.815	1	1	1	1	1	1	1	1	1
			80	32	1	1	1	1	1	1	1	1	1	1	888	0.682	1	1	1	1	1	1	1	1	1
		100	10	3	0.998	1	1	1	1	1	1	1	1	1	117	0.915	1	1	1	1	1	1	1	1	1
			20	8	1	1	1	1	1	1	1	1	1	1	438	0.860	1	1	1	1	1	1	1	1	1
			30	14	1	1	1	1	1	1	1	1	1	1	761	0.820	1	1	1	1	1	1	1	1	1
			50	66	1	1	1	1	1	1	1	1	1	1	974	0.729	1	1	1	1	1	1	1	1	1
			80	145	1	1	1	1	1	1	1	1	1	1	1,000	0.563	1	1	1	1	1	1	1	1	1
		200	10	13	0.999	1	1	1	1	1	1	1	1	1	452	0.913	1	1	1	1	1	1	1	1	1
			20	35	1	1	1	1	1	1	1	1	1	1	921	0.851	1	1	1	1	1	1	1	1	1
			30	90	1	1	1	1	1	1	1	1	1	1	993	0.780	1	1	1	1	1	1	1	1	1
			50	229	1	1	1	1	1	1	1	1	1	1	1,000	0.628	1	1	1	1	1	1	1	1	1
			80	525	1	1	1	1	1	1	1	1	1	1	1,000	0.460	1	1	1	1	1	1	1	1	1
	Med.	50	10	16	0.982	1	1	1	1	1	1	1	1	1	520	0.432	0.996	1	0.937	0.999	1	1	1	1	1
			20	67	0.978	1	1	1	1	1	1	1	1	1	909	0.134	0.968	1	0.913	0.999	1	1	1	1	1
			30	140	0.965	1	1	1	1	1	1	1	1	1	987	0.040	0.934	1	0.915	1	1	1	1	1	1
			50	378	0.955	1	1	1	1	1	1	1	1	1	1,000	0.001	0.810	1	0.885	1	1	1	1	1	1
			80	707	0.943	1	1	1	1	1	1	1	1	1	1,000	0.001	0.663	1	0.894	1	1	1	1	1	1
		100	10	52	0.995	1	1	1	1	1	1	1	1	1	934	0.167	0.997	1	0.942	0.999	1	1	1	1	1
			20	279	0.99	1	1	1	1	1	1	1	1	1	999	0.011	0.992	1	0.935	1	1	1	1	1	1
			30	532	0.983	1	1	1	1	1	1	1	1	1	1,000	0.000	0.963	1	0.949	1	1	1	1	1	1
			50	852	0.975	1	1	1	1	1	1	1	1	1	1,000	0.000	0.837	1	0.928	1	1	1	1	1	1
			80	986	0.949	1	1	1	1	1	1	1	1	1	1,000	0.000	0.697	1	0.948	1	1	1	1	1	1
		200	10	257	1	1	1	1	1	1	1	1	1	1	1,000	0.022	1	1	0.969	1	1	1	1	1	1
			20	752	1	1	1	1	1	1	1	1	1	1	1,000	0.000	0.996	1	0.973	1	1	1	1	1	1
			30	944	0.995	1	1	1	1	1	1	1	1	1	1,000	0.000	0.983	1	0.963	1	1	1	1	1	1
			50	998	0.991	1	1	1	1	1	1	1	1	1	1,000	0.000	0.888	1	0.981	1	1	1	1	1	1
			80	1,000	0.989	1	1	1	1	1	1	1	1	1	1,000	0.000	0.650	1	0.984	1	1	1	1	1	1
	High	50	10	189	0.831	1	1	0.989	1	1	1	1	1	1	982	0.016	0.629	0.999	0.213	0.615	0.988	1	1	0.804	1
			20	551	0.635	1	1	0.997	1	1	1	1	1	1	1,000	0.000	0.160	0.923	0.056	0.457	0.985	1	1	0.891	1
			30	812	0.440	0.999	1	0.999	1	1	1	1	1	1	1,000	0.000	0.061	0.745	0.033	0.377	0.983	1	1	0.917	1
			50	979	0.181	0.999	1	0.999	1	1	1	1	1	1	1,000	0.000	0.009	0.495	0.017	0.342	0.981	1	1	0.932	1
			80	1,000	0.099	0.999	1	1	1	1	1	1	1	1	1,000	0.000	0.001	0.281	0.010	0.299	0.994	1	1	0.965	1
		100	10	540	0.799	1	1	1	1	1	1	1	1	1	1,000	0.000	0.511	1	0.043	0.506	0.998	1	1	0.931	1
			20	955	0.408	1	1	1	1	1	1	1	1	1	1,000	0.000	0.039	0.961	0.006	0.35	0.996	1	1	0.961	1
			30	998	0.192	1	1	1	1	1	1	1	1	1	1,000	0.000	0.006	0.791	0.002	0.285	0.998	1	1	0.987	1
			50	1,000	0.048	1	1	1	1	1	1	1	1	1	1,000	0.000	0.001	0.391	0.002	0.216	0.997	1	1	0.986	1
			80	1,000	0.013	1	1	1	1	1	1	1	1	1	1,000	0.000	0.000	0.154	0.000	0.181	0.996	1	1	0.987	1
		200	10	956	0.722	1	1	1	1	1	1	1	1	1	1,000	0.000	0.404	1	0.002	0.396	1	1	1	0.984	1
			20	1,000	0.223	1	1	1	1	1	1	1	1	1	1,000	0.000	0.005	0.992	0.000	0.221	1	1	1	0.996	1
			30	1,000	0.061	1	1	1	1	1	1	1	1	1	1,000	0.000	0.000	0.832	0.000	0.130	1	1	1	0.998	1
			50	1,000	0.007	1	1	1	1	1	1	1	1	1	1,000	0.000	0.000	0.337	0.000	0.109	1	1	1	0.998	1
			80	1,000	0.000	1	1	1	1	1	1	1	1	1	1,000	0.000	0.000	0.058	0.000	0.081	1	1	1	0.999	1
3	Low	50	10	5	0.987	1	1	1	1	1	1	1	1	1	26	0.944	1	1	1	1	1	0.998	0.999	1	1
			20	2	0.999	1	1	1	1	1	1	1	1	1	112	0.895	1	1	1	1	1	1	1	1	1
			30	7	0.999	1	1	1	1	1	1	1	1	1	240	0.876	1	1	1	1	1	1	1	1	1
			50	8	1	1	1	1	1	1	1	1	1	1	601	0.789	1	1	1	1	1	1	1	1	1
			80	43	1	1	1	1	1	1	1	1	1	1	880	0.682	1	1	1	1	1	1	1	1	1
		100	10	5	0.997	1	1	1	1	1	1	1	1	1	112	0.918	1	1	1	1	1	1	1	1	1
			20	13	0.999	1	1	1	1	1	1	1	1	1	451	0.874	1	1	1	1	1	1	1	1	1
			30	22	1	1	1	1	1	1	1	1	1	1	728	0.827	1	1	1	1	1	1	1	1	1
			50	52	1	1	1	1	1	1	1	1	1	1	972	0.687	1	1	1	1	1	1	1	1	1
			80	111	1	1	1	1	1	1	1	1	1	1	1,000	0.548	1	1	1	1	1	1	1	1	1
		200	10	7	1	1	1	1	1	1	1	1	1	1	421	0.923	1	1	1	1	1	1	1	1	1
			20	35	1	1	1	1	1	1	1	1	1	1	926	0.848	1	1	1	1	1	1	1	1	1
			30	83	1	1	1	1	1	1	1	1	1	1	991	0.785	1	1	1	1	1	1	1	1	1
			50	222	1	1	1	1	1	1	1	1	1	1	1,000	0.648	1	1	1	1	1	1	1	1	1
			80	525	1	1	1	1	1	1	1	1	1	1	1,000	0.417	1	1	1	1	1	1	1	1	1
	Med.	50	10	12	0.986	1	1	1	1	1	1	1	1	1	426	0.517	0.998	1	0.947	1	1	1	1	1	1
			20	59	0.973	1	1	1	1	1	1	1	1	1	915	0.120	0.982	1	0.945	1	1	1	1	1	1
			30	132	0.967	1	1	1	1	1	1	1	1	1	988	0.034	0.917	1	0.911	0.999	1	1	1	1	1
			50	341	0.955	1	1	1	1	1	1	1	1	1	1,000	0.004	0.820	1	0.924	1	1	1	1	1	1
			80	687	0.940	1	1	1	1	1	1	1	1	1	1,000	0.000	0.636	0.999	0.913	1	1	1	1	1	1
		100	10	51	0.993	1	1	1	1	1	1	1	1	1	900	0.207	1	1	0.957	1	1	1	1	1	1
			20	240	0.992	1	1	1	1	1	1	1	1	1	999	0.008	0.991	1	0.950	1	1	1	1	1	1
			30	504	0.985	1	1	1	1	1	1	1	1	1	1,000	0.001	0.961	1	0.959	1	1	1	1	1	1
			50	835	0.966	1	1	1	1	1	1	1	1	1	1,000	0.000	0.816	1	0.948	1	1	1	1	1	1
			80	977	0.952	1	1	1	1	1	1	1	1	1	1,000	0.000	0.627	1	0.940	1	1	1	1	1	1
		200	10	229	0.996	1	1	1	1	1	1	1	1	1	999	0.020	1	1	0.981	1	1	1	1	1	1
			20	721	0.997	1	1	1	1	1	1	1	1	1	1,000	0.000	1	1	0.989	1	1	1	1	1	1
			30	925	0.999	1	1	1	1	1	1	1	1	1	1,000	0.000	0.993	1	0.989	1	1	1	1	1	1
			50	998	0.991	1	1	1	1	1	1	1	1	1	1,000	0.000	0.877	1	0.982	1	1	1	1	1	1
			80	1,000	0.974	1	1	1	1	1	1	1	1	1	1,000	0.000	0.640	1	0.988	1	1	1	1	1	1
	High	50	10	145	0.871	1	1	0.997	1	1	1	1	1	1	971	0.029	0.690	1	0.258	0.711	0.995	1	1	0.739	1
			20	524	0.640	1	1	1	1	1	1	1	1	1	999	0.001	0.193	0.944	0.080	0.521	0.993	1	1	0.815	1
			30	808	0.438	1	1	1	1	1	1	1	1	1	1,000	0.000	0.056	0.734	0.042	0.440	0.989	1	1	0.830	1
			50	968	0.212	1	1	1	1	1	1	1	1	1	1,000	0.000	0.007	0.450	0.019	0.366	0.990	1	1	0.867	1
			80	999	0.086	0.998	1	1	1	1	1	1	1	1	1,000	0.000	0.001	0.224	0.008	0.329	0.985	1	1	0.860	1
		100	10	478	0.837	1	1	1	1	1	1	1	1	1	1,000	0.001	0.614	1	0.085	0.636	1	1	1	0.865	1
			20	929	0.478	1	1	1	1	1	1	1	1	1	1,000	0.000	0.058	0.973	0.012	0.414	0.997	1	1	0.903	1
			30	992	0.238	1	1	1	1	1	1	1	1	1	1,000	0.000	0.006	0.784	0.004	0.328	1	1	1	0.920	1
			50	1,000	0.051	1	1	1	1	1	1	1	1	1	1,000	0.000	0.000	0.357	0.000	0.256	0.997	1	1	0.931	1
			80	1,000	0.010	1	1	1	1	1	1	1	1	1	1,000	0.000	0.000	0.125	0.001	0.234	1	1	1	0.945	1
		200	10	906	0.773	1	1	1	1	1	1	1	1	1	1,000	0.000	0.485	1	0.007	0.536	1	1	1	0.939	1
			20	1,000	0.267	1	1	1	1	1	1	1	1	1	1,000	0.000	0.002	0.997	0.000	0.276	1	1	1	0.980	1
			30	1,000	0.049	1	1	1	1	1	1	1	1	1	1,000	0.000	0.000	0.827	0.000	0.241	0.999	1	1	0.984	1
			50	1,000	0.006	1	1	1	1	1	1	1	1	1	1,000	0.000	0.000	0.280	0.000	0.154	1	1	1	0.992	1
			80	1,000	0.000	1	1	1	1	1	1	1	1	1	1,000	0.000	0.000	0.043	0.000	0.135	1	1	1	0.988	1

For *p*-values, the absolute number of how often *p* < 0.05 in the 1,000 simulations is given. The greyly highlighted cells represent power ≥80%.

Looking deeper into the conditions revealed—as expected—a marked effect of the strength of the bias on power. With low bias, only the χ^2^ statistic and the CFI cut-off at 0.99 were able to identify misfit given the bias. Furthermore, this power was conditional on sample size, number of re-testings and ICCs. Given low ICCs, the bias could not be detected reliably even in the largest data sets. Given medium ICCs, only the χ^2^ statistic had adequate power, and only with at least 100 subjects being tested at least 50 times. Given high ICCs, the χ^2^ statistic still outperformed the CFI ≥ 0.99, but here at least CFI ≥ 0.99 also had adequate power when 50 subjects or more were tested at least 80 times. The RMSEA, in contrast, did not have sufficient power (at best it rejected 2.1%).

As expected, a stronger bias was easier to detect. Again, the χ^2^ statistic performed best, followed by the CFI. The χ^2^ statistic had 100% power in all conditions with high ICCs and had close to maximum power for medium ICCs (only with 50 subjects being tested 10 times was there a lack of power). Results for CFI ≥ 0.99 were similar and with low ICCs CFI ≥ 0.99 failed to reject the misspecified models, while the χ^2^ statistic performed well as soon as at least 50 subjects were tested 80 times. Being more lenient with a CFI ≥ 0.95 led to much larger data sets being needed for adequate power, while the even more lenient CFI ≥ 0.90 only performed well with the largest sample size and highest retesting rate. Examining the RMSEA revealed that it only performed well with at least medium ICCs. Here, RMSEA < 0.06 performed well (i.e., it had adequate power in all conditions except that with the smallest data sets), while RMSEA < 0.08 needed 200 subjects tested 50 times or more, and RMSEA < 0.10 did not have sufficient power. For the medium ICC condition, these results are summarized in Table 4.

Another noteworthy finding was the performance of the SRMR-w given high ICCs. As stated, with low and medium ICCs the SRMR-w showed no power. With high ICCs, however, the already low power of SRMR-w < 0.08 decreased further with *in*creasing sample size.

### 3.5 Power: four biased items

Finally, we examined the conditions in which within-person measurement bias affected four items (*bias condition 4*). We report the percentage of how often fit-index fulfilled their cut-offs in Table 3 (the last third of Table A6 in Appendix covers all average model fit indices).

**Table 3:** Percentages of fit indices above/below their cut-off according to common SEM cut-offs for varying ICCs (low., med. = medium, high), number of participants (*n*), number of re-tests (*t*) and for the one nonuniform bias condition where four items were affected with low and high bias.

			Low											High										
			CFI				RMSEA			SRMR-b		SRMR-w		CFI				RMSEA			SRMR-b		SRMR-w	
ICC	*n*	*t*	*p*<0.05	≥0.99	≥0.95	≥0.90	<0.06	<0.08	<0.10	<0.08	<0.11	<0.08	<0.11	*p*<0.05	≥0.99	≥0.95	≥0.90	<0.06	<0.08	<0.10	<0.08	<0.11	<0.08	<0.11
Low	50	10	28	0.929	1	1	1	1	1	1	1	1	1	681	0.155	0.694	0.987	0.853	0.996	1	0.99	1	0.996	1
		20	75	0.920	1	1	1	1	1	1	1	1	1	980	0.008	0.396	0.972	0.824	0.997	1	1	1	1	1
		30	212	0.882	1	1	1	1	1	1	1	1	1	1,000	0.000	0.205	0.956	0.768	0.997	1	1	1	1	1
		50	527	0.794	1	1	1	1	1	1	1	1	1	1,000	0.000	0.044	0.880	0.755	1	1	1	1	1	1
		80	840	0.691	1	1	1	1	1	1	1	1	1	1,000	0.000	0.010	0.801	0.757	1	1	1	1	1	1
	100	10	105	0.922	1	1	1	1	1	1	1	1	1	985	0.013	0.521	0.997	0.838	0.999	1	1	1	1	1
		20	394	0.879	1	1	1	1	1	1	1	1	1	1,000	0.000	0.179	0.993	0.795	1	1	1	1	1	1
		30	721	0.802	1	1	1	1	1	1	1	1	1	1,000	0.000	0.045	0.988	0.774	1	1	1	1	1	1
		50	963	0.709	1	1	1	1	1	1	1	1	1	1,000	0.000	0.003	0.937	0.754	1	1	1	1	1	1
		80	999	0.535	1	1	1	1	1	1	1	1	1	1,000	0.000	0.000	0.810	0.754	1	1	1	1	1	1
	200	10	412	0.908	1	1	1	1	1	1	1	1	1	1,000	0.000	0.342	1	0.816	1	1	1	1	1	1
		20	888	0.856	1	1	1	1	1	1	1	1	1	1,000	0.000	0.043	0.999	0.812	1	1	1	1	1	1
		30	994	0.786	1	1	1	1	1	1	1	1	1	1,000	0.000	0.001	0.998	0.790	1	1	1	1	1	1
		50	1,000	0.640	1	1	1	1	1	1	1	1	1	1,000	0.000	0.000	0.974	0.804	1	1	1	1	1	1
		80	1,000	0.403	1	1	1	1	1	1	1	1	1	1,000	0.000	0.000	0.899	0.824	1	1	1	1	1	1
Med.	50	10	519	0.419	0.988	1	0.914	0.997	1	1	1	1	1	1,000	0.000	0.012	0.176	0.009	0.067	0.542	1	1	0.037	0.503
		20	927	0.098	0.944	1	0.900	1	1	1	1	1	1	1,000	0.000	0.000	0.008	0.000	0.011	0.448	1	1	0.026	0.590
		30	986	0.031	0.861	1	0.905	1	1	1	1	1	1	1,000	0.000	0.000	0.002	0.000	0.011	0.378	1	1	0.026	0.607
		50	1,000	0.003	0.729	1	0.907	1	1	1	1	1	1	1,000	0.000	0.000	0.000	0.000	0.004	0.338	1	1	0.018	0.614
		80	1,000	0.001	0.530	0.999	0.890	1	1	1	1	1	1	1,000	0.000	0.000	0.000	0.000	0.002	0.319	1	1	0.024	0.609
	100	10	929	0.137	0.999	1	0.928	1	1	1	1	1	1	1,000	0.000	0.000	0.033	0.000	0.005	0.457	1	1	0.006	0.579
		20	1,000	0.004	0.969	1	0.934	1	1	1	1	1	1	1,000	0.000	0.000	0.000	0.000	0.000	0.309	1	1	0.002	0.620
		30	1,000	0.000	0.879	1	0.921	1	1	1	1	1	1	1,000	0.000	0.000	0.000	0.000	0.000	0.284	1	1	0.002	0.622
		50	1,000	0.000	0.685	1	0.927	1	1	1	1	1	1	1,000	0.000	0.000	0.000	0.000	0.000	0.241	1	1	0.005	0.647
		80	1,000	0.000	0.455	1	0.928	1	1	1	1	1	1	1,000	0.000	0.000	0.000	0.000	0.000	0.251	1	1	0.001	0.670
	200	10	1,000	0.009	1	1	0.959	1	1	1	1	1	1	1,000	0.000	0.000	0.003	0.000	0.000	0.310	1	1	0.001	0.593
		20	1,000	0.000	0.995	1	0.973	1	1	1	1	1	1	1,000	0.000	0.000	0.000	0.000	0.000	0.209	1	1	0.000	0.661
		30	1,000	0.000	0.918	1	0.962	1	1	1	1	1	1	1,000	0.000	0.000	0.000	0.000	0.000	0.183	1	1	0.000	0.698
		50	1,000	0.000	0.676	1	0.979	1	1	1	1	1	1	1,000	0.000	0.000	0.000	0.000	0.000	0.131	1	1	0.000	0.677
		80	1,000	0.000	0.397	1	0.979	1	1	1	1	1	1	1,000	0.000	0.000	0.000	0.000	0.000	0.136	1	1	0.000	0.678
High	50	10	979	0.018	0.449	0.977	0.171	0.503	0.960	1	1	0.524	0.976	1,000	0.000	0.000	0.005	0.000	0.000	0.013	0.999	0.999	0.000	0.003
		20	999	0.001	0.090	0.733	0.051	0.364	0.949	1	1	0.604	0.994	1,000	0.000	0.000	0.000	0.000	0.000	0.000	1	1	0.000	0.000
		30	1,000	0.000	0.025	0.507	0.030	0.333	0.946	1	1	0.627	0.995	1,000	0.000	0.000	0.000	0.000	0.000	0.001	1	1	0.000	0.001
		50	1,000	0.000	0.005	0.230	0.011	0.256	0.934	1	1	0.658	0.996	1,000	0.000	0.000	0.000	0.000	0.000	0.000	0.998	0.998	0.000	0.000
		80	1,000	0.000	0.001	0.101	0.004	0.239	0.924	1	1	0.657	0.996	1,000	0.000	0.000	0.000	0.000	0.000	0.000	0.999	0.999	0.000	0.000
	100	10	1,000	0.000	0.248	0.996	0.024	0.344	0.988	1	1	0.603	0.998	1,000	0.000	0.000	0.000	0.000	0.000	0.000	1	1	0.000	0.000
		20	1,000	0.000	0.008	0.775	0.002	0.212	0.989	1	1	0.699	1	1,000	0.000	0.000	0.000	0.000	0.000	0.000	1	1	0.000	0.000
		30	1,000	0.000	0.001	0.402	0.001	0.192	0.978	1	1	0.654	1	1,000	0.000	0.000	0.000	0.000	0.000	0.000	1	1	0.000	0.000
		50	1,000	0.000	0.000	0.099	0.002	0.136	0.982	1	1	0.719	1	1,000	0.000	0.000	0.000	0.000	0.000	0.000	0.999	0.999	0.000	0.000
		80	1,000	0.000	0.000	0.039	0.000	0.138	0.968	1	1	0.726	1	1,000	0.000	0.000	0.000	0.000	0.000	0.000	0.999	0.999	0.000	0.000
	200	10	1,000	0.000	0.112	0.999	0.001	0.193	0.997	1	1	0.682	1	1,000	0.000	0.000	0.000	0.000	0.000	0.000	1	1	0.000	0.000
		20	1,000	0.000	0.000	0.773	0.000	0.091	0.997	1	1	0.729	1	1,000	0.000	0.000	0.000	0.000	0.000	0.000	1	1	0.000	0.000
		30	1,000	0.000	0.000	0.278	0.000	0.071	0.997	1	1	0.742	1	1,000	0.000	0.000	0.000	0.000	0.000	0.000	0.999	0.999	0.000	0.000
		50	1,000	0.000	0.000	0.024	0.000	0.042	0.998	1	1	0.782	1	1,000	0.000	0.000	0.000	0.000	0.000	0.000	1	1	0.000	0.000
		80	1,000	0.000	0.000	0.004	0.000	0.049	0.998	1	1	0.773	1	1,000	0.000	0.000	0.000	0.000	0.000	0.000	1	1	0.000	0.000

For *p*-values, the absolute number of how often *p* < 0.05 in the 1,000 simulations is given. The greyly highlighted cells represent power ≥ 80%.

For the low bias conditions, results were analogous to those of the conditions with two affected items, albeit more pronounced. The χ^2^ statistic performed best, while for low ICCs, reliable power was given only with larger data sets. By contrast, given medium ICCs, 50 subjects being tested 20 times was already sufficient to detect the within-person nonuniform bias; given high ICCs, the χ^2^ statistic had 100% power in nearly all conditions. The strictest CFI cut-off with CFI ≥ 0.99 followed nearly the same patterns as the χ^2^ statistic, with the notable difference that in the low ICC conditions CFI ≥ 0.99 did not reject the misspecified models. CFI ≥ 0.95 performed well only with high ICCs, while CFI ≥ 0.90 needed high ICCs and large data sets to be adequately powered. The RMSEA detected the bias only in high ICC conditions, and not at all when RMSEA < 0.10. SRMR-b and SRMR-w, in contrast, showed no power in any of these conditions. Again, SRMR-b < 0.08 in the high ICC conditions showed a decrease in power with *in*creasing data set size. Taken together, the χ^2^ statistic and CFI ≥ 0.99 did very well in identifying misfit given low-strength bias with at least medium ICCs; with high ICCs, CFI ≥ 0.95 and RMSEA < 06 were also adequately powered.

For the high-bias conditions, the χ^2^ statistic and CFI ≥ 0.99 performed extremely well. That is, only with the smallest data set of 50 subjects being tested 10 times did they not achieve adequate power. In nearly all other cases, the power was 100%. The more lenient CFI ≥ 0.95 was underpowered only when subjects were tested 10 times. Being even more lenient with CFI ≥ 0.90 resulted in poorer performance in the low ICC conditions, and in adequate power when ICCs were medium. Likewise, the RMSEA did not perform well for low ICC conditions, while for medium and high ICC conditions RMSEA < 0.08 was adequate. The more lenient RMSEA < 0.10 resulted in good performance in the high ICC conditions, but required 200 subjects being tested at least 30 times for adequate power. Results showed yet again that SRMR-b had no power, regardless of the cut-off used. SRMR-w had no power in the low ICC conditions, but with SRMR-w < 0.08 adequate power was achieved in the medium and high ICC conditions, while the more lenient SRMR-w < 0.11 was adequately powered only in the high ICC conditions. Taken together, the χ^2^ statistic and CFI ≥ 0.95 performed well in detecting the bias; as soon as ICCs were medium, CFI ≥ 0.90, RMSEA < 0.08 and SRMR-w < 0.08 were also adequately powered, while with high ICCs only SRMR-b was underpowered.

## 4 Discussion

In this simulation study we set out to determine how model fit in ML-SEM is affected by a within-person nonuniform measurement bias in intensive longitudinal data. Building upon factor loadings that previous studies have shown to be expected (Kim et al., 2016: λ between 0.41 and 0.83), we simulated a low within-person nonuniform measurement bias—factor loadings varying between the upper and the lower end of plausible factor loadings (λ = 0.7 vs. λ = 0.4)—and a high within-person nonuniform measurement bias—factor loadings varying between the upper end of plausible factor loading and being clearly lower than such bounds (λ = 0.7 vs. λ = 0.2). Before discussing the results in depth, we summarize which sample sizes and re-test frequencies were necessary for such a bias to cause sufficient misfit to be detectable in ML-SEM. We advice application of ML-SEM to intensive longitudinal data to fulfill the implied sample requirements, or to bare these limitations in mind while alternatively considering more exploratory approaches like Latent Markov Factor Analysis (Vogelsmeier et al., 2019). Our recommendations assume medium ICCs (*M* = 0.515; *SD* = 0.082) and high ICCs (M = 0.693; SD = 0.089), at least two out of six items being affected by the bias, and adequate power with power ≥0.80. In Table 4, we present our sample size recommendations for detecting both low- and high-strength bias under the specific conditions offered by this simulation study.

**Table 4:** Minimum sample size recommendations for identifying misfit in ML-SEM caused by within-person nonuniform measurement bias.

	Medium ICC (*M* = 0.515; *SD* = 0.082)		High ICC (*M* = 0.693; *SD* = 0.089)	
Cut-off	Low-strength bias	High-strength bias	Low-strength bias	High-strength bias
*p* < 0.05	*n* = 100, tested 50 times each *n* = 200, tested 30 times each	*n* = 50, tested 20 times each	*n* = 50, tested 30 times each *n* = 100, tested 20 times each *n* = 200, tested 10 times each	*n* = 50, tested 10 times each
CFI ≥ 0.99	Underpowered	*n* = 50, tested 20 times each	*n* = 50, tested 80 times each *n* = 100, tested 50 times each *n* = 200, tested 30 times each	*n* = 50, tested 10 times each
CFI ≥ 0.95	Underpowered	Underpowered	Underpowered	*n* = 50, tested 30 times each *n* = 100, tested 20 times each
CFI ≥ 0.90	Underpowered	Underpowered	Underpowered	*n* = 200, tested 80 times each
RMSEA < 0.06	Underpowered	Underpowered	Underpowered	*n* = 50, tested 20 times each
RMSEA < 0.08	Underpowered	Underpowered	Underpowered	*n* = 200, tested 30 times each
RMSEA < 0.10	Underpowered	Underpowered	Underpowered	Underpowered
SRMR-between < 0.08	Underpowered	Underpowered	Underpowered	Underpowered
SRMR-between < 0.11	Underpowered	Underpowered	Underpowered	Underpowered
SRMR-within < 0.08	Underpowered	Underpowered	Underpowered	Underpowered
SRMR-within < 0.11	Underpowered	Underpowered	Underpowered	Underpowered

Medium and high ICCs, at least two out of six items being affected by the bias and adequate power with 0.8 is assumed. The term “underpowered” is to be interpreted in the context of this simulation study's conditions; that is, adequate power could not be achieved when assessing 200 participants tested 80 times each.

Overall, we recommend evaluating model fit with the χ^2^ statistic, CFI and RMSEA. Even though standard software solutions readily provide SRMR specific for each level (SRMR-b and SRMR-w), our results discourage their usage. Furthermore, the χ^2^ statistic can be readily evaluated on a 5% level without needing to counter inflated Type I error rates, and smaller data sets should evaluate CFI and RMSEA at stricter cut-offs.

Note that as other simulation studies, these recommendations are derived from a single and idealized scenario (e.g., one factor with six items, a specific set of population parameters and misspecifications). It is therefore unwarranted to generalize these recommendations to all ML-SEM application. As scholars have already stressed, simulation studies can offer at best rules of thumbs but no golden rules (Marsh et al., 2004). Future simulation studies will need to evaluate how model fit evaluation in ML-SEM analyzing intensive longitudinal data will fare for different misspecification, different sets of items, multidimensional constructs and scales, missing data, and different estimators.

Despite these limitations, we recommend ML-SEM to scrutinize multiple-item questionnaires when used in intensive longitudinal data. A more systematic effort in doing so seems indicated given the lack of studies reporting psychometric properties of multiple-item questionnaires in intensive longitudinal data (Trull and Ebner-Priemer, 2020). After having thereby evaluated the tool of assessment, which can also be of theoretical interest if the nature of constructs is still disputed (e.g., factorial structure and relations between factors), researchers can proceed to analyses deepening insights into dynamical processes such as dynamic SEM (Asparouhov et al., 2018) or Latent Markov Factor Analysis (Vogelsmeier et al., 2019). A recent example of such a work flow can be found in research on machiavellianism and psychopathy (Walczak et al., 2026).

### 4.1 Type I error

We found ML-SEM Type I error to be well below the nominal level for the χ^2^ statistic and the fit indices across all conditions simulated, with the χ^2^ statistic and fit indices rejecting at worst 0.5% and 0.6% of the correctly specified models, respectively. As such, neither sample size (*n*), re-test frequencies (*t*), nor how pronounced the hierarchical data structure was (ICC) affected the Type I error rates. These findings contrast with the prior simulation studies of Hsu et al. (2015), which reported a slightly elevated Type I error rate (8.358% for the χ^2^ statistic). This is because Hsu et al. simulated an intentional misalignment of their population model and fitted the ML-SEM model with the commendable goal of simulating more realistic data. They increased noise in item responses by introducing two types of error variance to each item response, the second of which went beyond the traditional measurement error (cf. “minor-domain variance”, p. 201). Accordingly, Hsu et al. ascertained that their elevated Type I error rate can “also be viewed as a validation of the specification of the minor-domain variances in the population model.” (p. 204). Our simulation study, however, showed that when the population model was consistent with the fitted model, no elevated Type I error could be observed. At the same time, the below-nominal Type I error may also warrant future studies to explore the distributional properties of the χ^2^ statistic of ML-SEM, as not finding a nominal Type I error may indicate an underlying issue with the assumed distributional properties.

Turning to the fit indices, our results showed CFI, RMSEA, SRMR-b and SRMR-w performing very well when no within-person nonuniform measurement bias was present. As in previous simulation studies (Hsu et al., 2015), CFI was on average at 1, while RMSEA, SRMR-b and SRMR-w, approached 0.

On the one hand, these results corroborate the prior simulation literature and attest to the performance of the fit indices. On the other hand, taking these findings together with the below nominal Type I error rate of the χ^2^ statistic suggests that the tests may be overly conservative. It therefore stands to reason how model fit evaluation performs in less than ideal conditions, that is not in the ideal settings of simulation studies, and if in such settings global fit indices may be even less sensitive to multiple nuanced sources of misspecification overlapping.

We also wish to stress the utility of the χ^2^ statistic. Intensive longitudinal data sets are usually large. Consider, for example, that Rogoza et al. (2024) reported in their most comprehensive overview on assessing narcissism in intensive longitudinal data studies with 1,080, 3,979, and 10,830 data points [which aligns with the average of 4548.46 data points per participant reported by Kim et al. (2016) across 70 ML-SEM studies as well as Wrzus and Neubauer (2023) reporting 51.11 in 347 EMA studies]. Given the size of these data sets, researchers may be inclined to dismiss the χ^2^ statistic, assuming that in SEM it will reject too many fitting models. Given the Type I error rates we observed, we agree with Greiff and Heene (2017), who stressed the importance of the χ^2^ statistic, and we urge researchers not to dismiss the χ^2^ statistic too readily.

### 4.2 Power

Our results showed that if a within-person no-uniform measurement bias affected only one item (of six), then ML-SEM failed to exhibit noticeable model misfit. In line with this failure, the pattern of power results closely resembled that of the Type I error rates. Thus, neither increasing data set size nor increasing strength of the bias led to higher power. We therefore conclude that evaluating psychometric soundness of multiple-item questionnaires with ML-SEM cannot reliably preclude within-person nonuniform measurement bias that affects only one item. At this point, prospective simulation studies may want to tackle the question of how practically relevant a distortion of just one item may be and suggest practical guidelines for intensive longitudinal data, addressing when measurement bias can lead to substantial distortions (cf. for single-level SEM: Lai et al., 2017).

Unlike for the condition with only one biased item, our results showed that when within-person nonuniform measurement bias affected two or more items (of six), ML-SEM showed model misfit. In this context, the χ^2^ statistic and CFI outperformed the other modes of model evaluation (RMSEA, SRMR-b, SRMR-w). These results depended on bias strength and size of the data set, which agrees with previous ML-SEM simulation studies (e.g., Hsu et al., 2015) and single-level simulation studies (e.g., Hu and Bentler, 1999). We want to stress that, given a bias that affects enough items sufficiently strongly, especially the χ^2^ statistic reached a power of 100% already for the smallest sample sizes and the smallest number of re-testings. Like other scholars (e.g., Greiff and Heene, 2017), we therefore recommend that researchers pay attention to the χ^2^ statistic not only due to its low Type I error (cf. results on Type I error rates above), but also due to its sensitivity in detecting model misspecification.

As power was dependent on bias strength and data set size, even RMSEA was able to achieve adequate power with strong bias and sufficiently large data sets. In the conditions where RMSEA was affected, < 0.06 outperformed the other cut-offs for RMSEA. Our results are therefore in line with previous studies that warned of RMSEA not being sufficiently sensitive (Heene et al., 2011; Hsu et al., 2015). If researchers intend to use RMSEA, we recommend adhering to the strictest cut-off of 0.06.

Some of our SRMR-b and SRMR-w results are in line with those of previous ML-SEM simulation studies, but some also shed new light on SRMRs in the multilevel context. First, we found SRMR-b to be severely underpowered in all conditions. It has already been noted by other scholars that this fit index is more sensitive to model misspecification at the *between* than the *within* level (Hsu et al., 2015; Kim et al., 2016). As our bias was introduced *within person*, the poor performance of the SRMR-b was expected, but not its degree: the SRMR-b did not achieve any power in any condition. We therefore caution researchers against drawing any conclusion related to within-person nonuniform measurement bias on the basis of SRMR-b.

Second, we found the SRMR-w to perform fairly well only with the strongest bias present, and even then a strict cut-off was necessary. Contrary to what one might expect, the SRMR-w had a unique relation with the size of the data set: In certain conditions it showed a decrease in power with *in*creasing size of the data sets. While these results contradict general considerations for the relation between sample size and power, they have been observed before: Marsh et al. (2004) found that the single-level SRMR had less power with greater sample sizes in certain conditions, namely a complex structure with cross-loadings present, and this effect became apparent only when SRMR was already underpowered. It would seem that SRMR-w either performs well or fails catastrophically. We speculate that this may be due to the case that SRMRs are oversimplified. At the core, SRMRs compare correlations. However, correlations are easily distorted by outliers (Cousineau and Chartier, 2010) and distributional properties (Bishara and Hittner, 2015), which may play a different role depending on the sample size. Accordingly alternative SRMRs could be considered that are conditional on sample sizes or implement other coefficients to gauge the strength of relations between variables. Given this counter-intuitive relation between sample size and power, we caution researchers against relying too much on the SRMR-w despite its easy availability in standard software solutions.

Finally, we also found that item ICCs strongly influenced the detection of model misspecification. First, these effects could already be seen on the ICCs at the item level, as items affected by within-person nonuniform measurement bias exhibited consistently lower ICCs than unaffected items. These results need to be viewed in light of what an ICC assesses. Applying Equation 6, which shows that an ICC is the ratio of variance of the higher level to the entire variance, yields for the two-level intensive longitudinal case


$$\begin{array}{l} ICC=\frac{\Phi_{b}+\theta \varepsilon_{b}}{\Phi_{b}+\theta \varepsilon_{b}+\Phi_{w}+\theta \varepsilon_{w}} \end{array}$$ (7)

where Φ_b_ and Φ_w_ denote the latent variances and Θε_b_ and Θε_w_ denote the residuals at the respective level. Note that in latent variable modeling, variances on the manifest level are explained by the variances of the latent trait and the residuals. As Equation 7 shows, the ICC is affected by the systematic variances on both levels (Φ_b_ and Φ_w_) and by the variances of the residuals Θε_b_ and Θε_w_ which engulf the systematic unexplained variance and the unsystematic variances. A within-person nonuniform measurement bias leads to lower factor loadings, which goes hand in hand with increased variances of the residuals on the within-person level, that is an increase in Θε_w_ by equal systematic variances and equal variances of residuals on the between-person level. Accordingly, a drop in the ICC can be observed for these items that are affected by the bias. This implies that for evaluating multiple-item questionnaires, researcher can in a first step inspect the ICCs of all items. Items that differ in their ICCs while prior work does not suggest that they differ in the ability to differentiate between individuals with higher and lower traits may be a first indicator of a within-person nonuniform measurement bias. Such a first examination would also be encouraged by the fact that ICCs are easily computable and implemented in all software solution that can deal with hierarchical data structures.

Furthermore, we found that the ICC magnitude had a similar effect as bias strength, and that higher ICCs resulted in bias being detected more easily. This finding contrasts with those of previous ML-SEM simulation studies, which concluded trivial influence of ICCs (Hsu et al., 2015). This difference may be due to the range of ICCs and the nature of the model misspecifications simulated. The highest ICCs in the study by Hsu et al. corresponded to our medium ICCs (0.5 vs. an average ICC of 0.515 in our study). Accordingly, the effects of this design factor may not be fully comparable between these simulation studies. Furthermore, this discrepancy may also stress that model misspecification at the *within* level (i.e., our within-person nonuniform measurement bias) is affected by how pronounced the hierarchical data structure is. In support of this, consider that Hsu et al. did not introduce model misspecification within their clusters.

As our study is the first of its kind to simulate within-person nonuniform measurement bias, the ICC effect we observed may offer a unique view compared to prior simulation studies. Consider that the ICC effect in our study implies: the more pronounced interindividual differences (*SD*_b_ leading to an increase in ICCs), the more pronounced the effects of intraindividual differences. This aligns with the notation that in current psychological research that addressing intraindividual differences relies on interindividual differences (Krammer, 2025). These findings have the direct implication for future person-oriented research and indicate that we can more easily study intraindividual differences by maximizing the interindividual differences between our participants. Phrased in the opposing way, if all people are overall the same then there is no systematic variance that a multilevel structure would need (or could) heed. It follows that phenomena like the within-person measurement bias in this simulations study stemming from differences between measurement occasions of the same people, can be treated as person-independent differences, that is like independent data points in a single-level analyses. Future studies would need to address if this effect is specific to a within-person nonuniform measurement bias as it affects factor loadings on the within-person level, of such an increase in power can also be observed for other forms of bias. In doing so, future studies should also determine how power for detecting different kinds of bias in the framework of structural equation modeling can be gauged.

### 4.3 Limitations and conclusion

While we did implement 450 conditions, our findings were derived under specific conditions, and it stands to reason how our findings can be transferred to other settings. We did not simulate a variety of test lengths and factor loadings (e.g., Heene et al., 2011) or multiple factors and cross-loadings (e.g., Hsu et al., 2015). We also did not simulate different distributional properties (e.g., Hu and Bentler, 1999). This was most likely the reason that we ran into no convergence issues. Our simulation study can therefore not inform practitioners about how to handle convergence issues or when convergence issues are likely to occur. Factor loadings could also be varied in the bias conditions, therefore offering a more nuanced and stepwise take on how much nonuniform measurement bias is introduced by varying λ_wi_. Such future studies should also include considerations of what a measurement bias means on a practical level. Doing so would also stress that scholars have pointed out that practical significance of bias above and beyond significant changes in model fits. We also simulated a fixed ratio of how many measurement points are affected by the within-person nonuniform measurement bias, that is, half of the measurement points were affected in our bias conditions for the entire sample. Other study may vary this ratio as a further mean of varying bias strength, and may also address systematic effects such as effects of the week days (e.g., every 5^th^ out of seven measurement point is biased to simulated a “Thank God it's Friday” effect: Camerman et al., 2024). When considering different patterns of bias, future simulations studies may also implement more person-specific effects, for example, different within-person measurement bias up until the granularity of every individual in a sample. While we believe that this might exceed what quantitative psychological methods can handle, going stepwise in this direction may address how general insights are and how strongly general insights may be related to the individual (for similar lines of reasoning cf. Bakan, 1955; Krammer, 2025; Renner et al., 2020). Either way, we need to acknowledge the limitation that in this simulation study the within-person nonuniform measurement bias can also be seen as a between-person effect, constituted by interindividual differences within intraindividual processes across time. Along this line, a measurement bias may also be induced by continuous variables varying over time, with effects more nuanced than the dichotomous bias vs. no-bias. Future simulation studies may therefore also induce measurement bias in such a nuanced fashion. We also did not simulate other types of within-person model misspecification, such as local dependencies *within person*. Also, since our study is the first of its kind, we evaluated commonly used cut-off criteria. Other avenues worth exploring may be to evaluate model fit without relying on cut-offs (cf. criticism of cut-offs in Marsh et al., 2004). One could do so, for example, by strengthening graphical model inspections.

Further, we simulated only two levels. For intensive longitudinal data, it would be conceivable to introduce more levels. One could nest participants' responses within days of testing, and in turn within participants; or one could introduce a nesting structure based on participants' responses before and after major life events (cf. effects of life events: Bleidorn et al., 2018). These and related thoughts about a more complex nesting structure have in common the underlying question of what level of aggregation would be the most appropriate for a given study and its aims (cf. Krammer, 2023). Intensive longitudinal data raise this question very directly, as researchers must justify why less intensive modes of data collection are not sufficient.

To the best of our knowledge, our study is the first to address within-person measurement bias in the ML-SEM framework. It is therefore also the first to scrutinize a confirmatory approach to addressing psychometrical evaluations of multiple-item questionnaires in intensive longitudinal data. Unique to this approach, as opposed to methods geared toward parameter estimation or methods exploring exploratory avenues, is the evaluation of model fit in the SEM framework. As our study attested to the performance of ML-SEM, it sheds light on how we can be aware of nonuniform measurement bias when dealing with studying intraindividual differences. While we did so motivated by ecological momentary assessment, our findings can be readily transferred to other research settings that yield the same data structure (e.g., ambulatory assessment, daily diary studies, experience sampling methods, and hierarchical data structures in general).

#### Open science statement

We provide Open Data and Open Materials. All data and corresponding R-scripts are freely available at https://osf.io/79hp5/, as is the online supplementary material (Appendix). Since this was a simulation study, we did not preregister our hypotheses.

## Data Availability

The datasets presented in this study can be found in the online repositories OSF under https://osf.io/79hp5/.

## Footnotes

1 Please note that these addressed “measurement” and “bias” in the broad sense and also found, for example, titles with “questionnaire measurement” or “attentional bias”.

2 A uniform measurement bias would be given in (3) if τ_i_ varied systematically between subgroups of respondents.

3 Trull and Ebner-Priemer (2020, Table 1) found that only 30% of the studies surveyed “Report psychometric properties of items in the current EMA-study (between and within person), as well as the origin of the items”.
