# Supplementary material for: When our measurements are different every day: an ML-SEM simulation study on within-person nonuniform measurement bias in intensive longitudinal data
Source: Front Psychol. 2026 Apr 23;17:1624037. doi: 10.3389/fpsyg.2026.1624037 (PMC13149404; doi:10.3389/fpsyg.2026.1624037)
Supplement: Supplementary file 1 [file Table_1.docx]

# Appendix

# Results: Intraclass correlations

Table A1

Average intraclass correlations (ICC) of all items (i01 through i06) for varying between-person differences (*SD*_b_), number of participants (n), number of re-tests (t) and for the four different nonuniform bias conditions (Bias) with low/high bias. Please note that cells highlighted in bold are the ICCs of items affected by the bias.

|  |  |  |  | Low | | | | | | High | | | | | |
| --- | --- | --- | --- | --- | --- | --- | --- | --- | --- | --- | --- | --- | --- | --- | --- |
| Bias | *SD*_b_ | n | t | i01 | i02 | i03 | i04 | i05 | i06 | i01 | i02 | i03 | i04 | i05 | i06 |
| None | 1 | 50 | 10 | .244 | .243 | .244 | .242 | .244 | .243 | .247 | .246 | .247 | .246 | .246 | .246 |
| (=Type I Error) |  |  | 20 | .242 | .243 | .243 | .242 | .244 | .243 | .245 | .247 | .245 | .246 | .246 | .246 |
|  |  |  | 30 | .244 | .245 | .245 | .244 | .246 | .245 | .248 | .248 | .248 | .247 | .247 | .247 |
|  |  |  | 50 | .246 | .245 | .245 | .245 | .246 | .245 | .247 | .246 | .246 | .246 | .246 | .246 |
|  |  |  | 80 | .245 | .244 | .245 | .245 | .245 | .245 | .246 | .245 | .246 | .246 | .246 | .246 |
|  |  | 100 | 10 | .248 | .247 | .248 | .247 | .247 | .249 | .246 | .249 | .247 | .247 | .247 | .247 |
|  |  |  | 20 | .246 | .246 | .246 | .246 | .246 | .245 | .247 | .247 | .248 | .247 | .247 | .246 |
|  |  |  | 30 | .246 | .246 | .246 | .246 | .246 | .246 | .247 | .246 | .247 | .246 | .246 | .246 |
|  |  |  | 50 | .246 | .245 | .245 | .245 | .245 | .245 | .245 | .246 | .246 | .246 | .245 | .246 |
|  |  |  | 80 | .245 | .246 | .245 | .246 | .246 | .245 | .246 | .246 | .246 | .246 | .246 | .246 |
|  |  | 200 | 10 | .248 | .247 | .247 | .247 | .247 | .247 | .247 | .247 | .246 | .247 | .247 | .246 |
|  |  |  | 20 | .246 | .246 | .246 | .246 | .246 | .245 | .247 | .247 | .248 | .248 | .248 | .247 |
|  |  |  | 30 | .246 | .247 | .246 | .246 | .246 | .246 | .246 | .246 | .246 | .246 | .246 | .246 |
|  |  |  | 50 | .247 | .247 | .247 | .247 | .248 | .247 | .246 | .246 | .246 | .246 | .246 | .246 |
|  |  |  | 80 | .247 | .247 | .247 | .246 | .247 | .247 | .246 | .247 | .246 | .246 | .246 | .246 |
|  | 2 | 50 | 10 | .566 | .566 | .566 | .564 | .566 | .566 | .560 | .559 | .559 | .559 | .558 | .559 |
|  |  |  | 20 | .561 | .561 | .561 | .561 | .561 | .560 | .564 | .564 | .563 | .564 | .563 | .564 |
|  |  |  | 30 | .562 | .562 | .562 | .562 | .561 | .562 | .562 | .562 | .562 | .562 | .562 | .562 |
|  |  |  | 50 | .562 | .562 | .562 | .562 | .562 | .562 | .563 | .563 | .563 | .562 | .563 | .563 |
|  |  |  | 80 | .562 | .562 | .562 | .562 | .562 | .562 | .561 | .562 | .561 | .562 | .562 | .562 |
|  |  | 100 | 10 | .561 | .562 | .562 | .562 | .563 | .562 | .565 | .564 | .565 | .565 | .564 | .565 |
|  |  |  | 20 | .562 | .562 | .562 | .562 | .563 | .563 | .565 | .565 | .565 | .565 | .565 | .564 |
|  |  |  | 30 | .563 | .563 | .563 | .563 | .563 | .563 | .566 | .566 | .566 | .566 | .566 | .566 |
|  |  |  | 50 | .567 | .566 | .567 | .567 | .567 | .567 | .566 | .566 | .566 | .566 | .566 | .566 |
|  |  |  | 80 | .565 | .565 | .565 | .565 | .565 | .565 | .563 | .562 | .562 | .563 | .562 | .563 |
|  |  | 200 | 10 | .567 | .566 | .567 | .567 | .566 | .567 | .566 | .567 | .567 | .567 | .566 | .567 |
|  |  |  | 20 | .566 | .566 | .566 | .566 | .566 | .566 | .566 | .566 | .566 | .566 | .566 | .566 |
|  |  |  | 30 | .566 | .566 | .566 | .566 | .566 | .566 | .566 | .566 | .566 | .566 | .566 | .566 |
|  |  |  | 50 | .566 | .566 | .566 | .566 | .566 | .566 | .566 | .566 | .566 | .566 | .566 | .566 |
|  |  |  | 80 | .566 | .566 | .566 | .566 | .566 | .566 | .567 | .567 | .566 | .567 | .567 | .567 |
|  | 3 | 50 | 10 | .740 | .741 | .740 | .741 | .740 | .741 | .741 | .742 | .742 | .742 | .742 | .741 |
|  |  |  | 20 | .741 | .741 | .740 | .741 | .741 | .741 | .740 | .740 | .740 | .740 | .740 | .740 |
|  |  |  | 30 | .741 | .741 | .741 | .741 | .741 | .741 | .741 | .741 | .741 | .741 | .741 | .742 |
|  |  |  | 50 | .743 | .743 | .743 | .743 | .743 | .742 | .741 | .741 | .741 | .742 | .741 | .741 |
|  |  |  | 80 | .740 | .741 | .741 | .741 | .741 | .741 | .740 | .740 | .740 | .740 | .740 | .741 |
|  |  | 100 | 10 | .744 | .744 | .744 | .745 | .745 | .744 | .745 | .745 | .745 | .745 | .745 | .745 |
|  |  |  | 20 | .744 | .744 | .744 | .744 | .744 | .744 | .744 | .744 | .743 | .743 | .744 | .744 |
|  |  |  | 30 | .743 | .744 | .743 | .744 | .743 | .744 | .745 | .744 | .744 | .745 | .745 | .745 |
|  |  |  | 50 | .745 | .745 | .745 | .745 | .745 | .745 | .745 | .744 | .744 | .744 | .744 | .744 |
|  |  |  | 80 | .745 | .744 | .744 | .744 | .744 | .744 | .744 | .745 | .744 | .744 | .745 | .745 |
|  |  | 200 | 10 | .745 | .746 | .746 | .746 | .746 | .746 | .746 | .746 | .746 | .746 | .747 | .746 |
|  |  |  | 20 | .744 | .745 | .744 | .745 | .745 | .745 | .745 | .745 | .745 | .745 | .745 | .745 |
|  |  |  | 30 | .747 | .747 | .746 | .746 | .747 | .747 | .747 | .746 | .746 | .746 | .746 | .746 |
|  |  |  | 50 | .745 | .745 | .745 | .745 | .745 | .745 | .745 | .745 | .745 | .745 | .745 | .745 |
|  |  |  | 80 | .744 | .744 | .744 | .744 | .744 | .744 | .745 | .745 | .745 | .745 | .745 | .745 |
| 1 | 1 | 50 | 10 | .244 | .243 | .244 | .243 | .244 | **.177** | .245 | .245 | .245 | .245 | .244 | **.125** |
|  |  |  | 20 | .245 | .245 | .245 | .245 | .245 | **.182** | .244 | .244 | .246 | .245 | .246 | **.128** |
|  |  |  | 30 | .244 | .245 | .245 | .244 | .246 | **.180** | .245 | .246 | .245 | .247 | .246 | **.130** |
|  |  |  | 50 | .241 | .243 | .243 | .242 | .242 | **.179** | .246 | .245 | .246 | .246 | .246 | **.130** |
|  |  |  | 80 | .245 | .246 | .246 | .246 | .245 | **.182** | .246 | .246 | .246 | .246 | .246 | **.131** |
|  |  | 100 | 10 | .247 | .246 | .246 | .247 | .246 | **.181** | .249 | .247 | .247 | .247 | .247 | **.127** |
|  |  |  | 20 | .245 | .245 | .245 | .245 | .245 | **.181** | .246 | .247 | .247 | .246 | .246 | **.129** |
|  |  |  | 30 | .247 | .247 | .247 | .247 | .247 | **.182** | .245 | .244 | .245 | .245 | .244 | **.130** |
|  |  |  | 50 | .246 | .246 | .246 | .246 | .246 | **.182** | .246 | .246 | .246 | .247 | .247 | **.131** |
|  |  |  | 80 | .248 | .248 | .248 | .248 | .248 | **.184** | .245 | .245 | .245 | .245 | .245 | **.131** |
|  |  | 200 | 10 | .247 | .247 | .246 | .246 | .246 | **.181** | .246 | .246 | .246 | .246 | .246 | **.128** |
|  |  |  | 20 | .246 | .246 | .246 | .246 | .246 | **.182** | .247 | .247 | .247 | .247 | .248 | **.129** |
|  |  |  | 30 | .247 | .247 | .247 | .246 | .247 | **.182** | .247 | .247 | .247 | .247 | .247 | **.131** |
|  |  |  | 50 | .248 | .248 | .248 | .249 | .248 | **.184** | .247 | .248 | .247 | .248 | .247 | **.132** |
|  |  |  | 80 | .246 | .246 | .246 | .246 | .246 | **.182** | .247 | .248 | .247 | .248 | .247 | **.132** |
|  | 2 | 50 | 10 | .562 | .560 | .561 | .562 | .562 | **.452** | .561 | .560 | .560 | .559 | .559 | **.331** |
|  |  |  | 20 | .565 | .565 | .566 | .565 | .565 | **.458** | .565 | .565 | .565 | .564 | .565 | **.341** |
|  |  |  | 30 | .562 | .562 | .563 | .563 | .563 | **.455** | .562 | .560 | .561 | .562 | .561 | **.340** |
|  |  |  | 50 | .562 | .561 | .561 | .561 | .562 | **.455** | .562 | .562 | .562 | .561 | .562 | **.343** |
|  |  |  | 80 | .561 | .561 | .561 | .561 | .561 | **.454** | .562 | .562 | .562 | .562 | .562 | **.343** |
|  |  | 100 | 10 | .564 | .564 | .562 | .563 | .563 | **.453** | .565 | .566 | .566 | .566 | .565 | **.337** |
|  |  |  | 20 | .565 | .565 | .564 | .564 | .565 | **.456** | .565 | .565 | .566 | .566 | .565 | **.341** |
|  |  |  | 30 | .563 | .563 | .563 | .564 | .563 | **.455** | .564 | .564 | .564 | .564 | .564 | **.341** |
|  |  |  | 50 | .567 | .566 | .566 | .567 | .567 | **.459** | .566 | .566 | .566 | .566 | .566 | **.345** |
|  |  |  | 80 | .566 | .566 | .566 | .566 | .566 | **.459** | .566 | .566 | .566 | .566 | .566 | **.346** |
|  |  | 200 | 10 | .567 | .566 | .566 | .566 | .566 | **.455** | .565 | .565 | .565 | .566 | .566 | **.335** |
|  |  |  | 20 | .566 | .566 | .566 | .566 | .566 | **.458** | .567 | .567 | .567 | .567 | .567 | **.342** |
|  |  |  | 30 | .566 | .566 | .566 | .566 | .566 | **.458** | .566 | .566 | .566 | .566 | .566 | **.343** |
|  |  |  | 50 | .566 | .566 | .567 | .567 | .567 | **.459** | .568 | .567 | .568 | .567 | .568 | **.346** |
|  |  |  | 80 | .567 | .567 | .567 | .567 | .567 | **.460** | .566 | .566 | .566 | .566 | .566 | **.345** |
|  | 3 | 50 | 10 | .740 | .740 | .740 | .739 | .741 | **.628** | .740 | .741 | .742 | .740 | .741 | **.476** |
|  |  |  | 20 | .741 | .741 | .741 | .740 | .741 | **.633** | .742 | .742 | .742 | .742 | .742 | **.487** |
|  |  |  | 30 | .745 | .745 | .745 | .745 | .745 | **.637** | .740 | .741 | .740 | .740 | .740 | **.488** |
|  |  |  | 50 | .742 | .742 | .742 | .742 | .742 | **.634** | .743 | .743 | .743 | .743 | .742 | **.493** |
|  |  |  | 80 | .744 | .744 | .743 | .743 | .743 | **.636** | .740 | .739 | .739 | .740 | .740 | **.491** |
|  |  | 100 | 10 | .745 | .744 | .745 | .744 | .744 | **.632** | .746 | .746 | .745 | .745 | .745 | **.481** |
|  |  |  | 20 | .745 | .745 | .745 | .745 | .745 | **.636** | .744 | .744 | .744 | .744 | .744 | **.488** |
|  |  |  | 30 | .744 | .744 | .744 | .744 | .744 | **.635** | .745 | .745 | .745 | .745 | .745 | **.491** |
|  |  |  | 50 | .745 | .745 | .745 | .745 | .746 | **.638** | .744 | .744 | .744 | .744 | .744 | **.494** |
|  |  |  | 80 | .744 | .744 | .744 | .744 | .744 | **.637** | .744 | .744 | .744 | .744 | .743 | **.494** |
|  |  | 200 | 10 | .746 | .747 | .746 | .746 | .746 | **.634** | .745 | .745 | .745 | .745 | .745 | **.480** |
|  |  |  | 20 | .746 | .746 | .746 | .746 | .746 | **.636** | .745 | .745 | .745 | .745 | .745 | **.489** |
|  |  |  | 30 | .746 | .745 | .745 | .745 | .746 | **.637** | .745 | .745 | .745 | .745 | .745 | **.492** |
|  |  |  | 50 | .746 | .746 | .746 | .746 | .746 | **.638** | .745 | .745 | .745 | .745 | .745 | **.494** |
|  |  |  | 80 | .746 | .746 | .746 | .746 | .746 | **.638** | .745 | .745 | .745 | .745 | .745 | **.495** |
| 2 | 1 | 50 | 10 | .241 | .242 | .243 | .244 | **.179** | **.177** | .244 | .243 | .244 | .244 | **.128** | **.125** |
|  |  |  | 20 | .245 | .245 | .245 | .245 | **.181** | **.181** | .245 | .245 | .245 | .246 | **.130** | **.129** |
|  |  |  | 30 | .246 | .248 | .246 | .246 | **.182** | **.181** | .246 | .245 | .245 | .245 | **.130** | **.130** |
|  |  |  | 50 | .244 | .244 | .245 | .246 | **.182** | **.181** | .244 | .244 | .243 | .243 | **.129** | **.130** |
|  |  |  | 80 | .246 | .246 | .245 | .246 | **.182** | **.182** | .245 | .245 | .244 | .245 | **.130** | **.131** |
|  |  | 100 | 10 | .246 | .245 | .245 | .244 | **.179** | **.180** | .244 | .244 | .243 | .245 | **.126** | **.126** |
|  |  |  | 20 | .244 | .244 | .245 | .244 | **.179** | **.181** | .245 | .244 | .245 | .246 | **.128** | **.128** |
|  |  |  | 30 | .245 | .245 | .246 | .245 | **.181** | **.181** | .247 | .247 | .247 | .247 | **.131** | **.131** |
|  |  |  | 50 | .248 | .248 | .248 | .249 | **.184** | **.184** | .246 | .246 | .246 | .246 | **.131** | **.131** |
|  |  |  | 80 | .248 | .248 | .248 | .248 | **.184** | **.183** | .247 | .247 | .247 | .247 | **.132** | **.132** |
|  |  | 200 | 10 | .247 | .247 | .247 | .246 | **.181** | **.182** | .245 | .245 | .245 | .244 | **.127** | **.127** |
|  |  |  | 20 | .247 | .248 | .247 | .247 | **.182** | **.182** | .247 | .248 | .247 | .247 | **.129** | **.130** |
|  |  |  | 30 | .248 | .248 | .248 | .248 | **.183** | **.183** | .247 | .247 | .246 | .246 | **.131** | **.131** |
|  |  |  | 50 | .247 | .247 | .247 | .247 | **.183** | **.183** | .246 | .246 | .246 | .246 | **.131** | **.131** |
|  |  |  | 80 | .246 | .247 | .247 | .247 | **.182** | **.182** | .247 | .246 | .247 | .247 | **.131** | **.131** |
|  | 2 | 50 | 10 | .561 | .560 | .560 | .560 | **.451** | **.450** | .562 | .564 | .562 | .563 | **.332** | **.333** |
|  |  |  | 20 | .565 | .564 | .564 | .564 | **.455** | **.456** | .561 | .562 | .561 | .562 | **.338** | **.339** |
|  |  |  | 30 | .560 | .559 | .559 | .560 | **.454** | **.452** | .561 | .561 | .561 | .561 | **.340** | **.341** |
|  |  |  | 50 | .561 | .561 | .560 | .561 | **.453** | **.453** | .561 | .561 | .561 | .561 | **.342** | **.342** |
|  |  |  | 80 | .561 | .561 | .561 | .561 | **.455** | **.455** | .563 | .563 | .563 | .563 | **.345** | **.344** |
|  |  | 100 | 10 | .567 | .566 | .567 | .566 | **.455** | **.456** | .565 | .566 | .565 | .566 | **.335** | **.335** |
|  |  |  | 20 | .564 | .564 | .564 | .564 | **.455** | **.455** | .565 | .565 | .565 | .565 | **.340** | **.341** |
|  |  |  | 30 | .566 | .566 | .566 | .566 | **.459** | **.458** | .564 | .563 | .564 | .564 | **.342** | **.342** |
|  |  |  | 50 | .564 | .565 | .565 | .565 | **.457** | **.457** | .566 | .566 | .566 | .565 | **.345** | **.344** |
|  |  |  | 80 | .565 | .565 | .565 | .565 | **.458** | **.457** | .564 | .564 | .564 | .564 | **.344** | **.344** |
|  |  | 200 | 10 | .567 | .568 | .567 | .567 | **.456** | **.456** | .566 | .566 | .565 | .567 | **.335** | **.334** |
|  |  |  | 20 | .568 | .568 | .568 | .568 | **.459** | **.459** | .568 | .568 | .568 | .568 | **.342** | **.343** |
|  |  |  | 30 | .567 | .567 | .567 | .567 | **.459** | **.459** | .567 | .567 | .567 | .567 | **.344** | **.344** |
|  |  |  | 50 | .567 | .567 | .567 | .567 | **.460** | **.459** | .567 | .567 | .567 | .567 | **.345** | **.345** |
|  |  |  | 80 | .565 | .565 | .565 | .565 | **.458** | **.457** | .567 | .567 | .567 | .567 | **.346** | **.346** |
|  | 3 | 50 | 10 | .744 | .744 | .743 | .744 | **.633** | **.631** | .744 | .743 | .742 | .743 | **.479** | **.479** |
|  |  |  | 20 | .742 | .742 | .742 | .741 | **.632** | **.632** | .742 | .742 | .743 | .742 | **.487** | **.488** |
|  |  |  | 30 | .742 | .741 | .741 | .741 | **.632** | **.633** | .743 | .743 | .743 | .743 | **.490** | **.490** |
|  |  |  | 50 | .745 | .745 | .745 | .745 | **.637** | **.638** | .740 | .740 | .741 | .740 | **.491** | **.491** |
|  |  |  | 80 | .741 | .741 | .742 | .742 | **.634** | **.634** | .740 | .740 | .740 | .741 | **.492** | **.492** |
|  |  | 100 | 10 | .745 | .745 | .745 | .745 | **.633** | **.633** | .745 | .745 | .744 | .745 | **.480** | **.480** |
|  |  |  | 20 | .745 | .745 | .745 | .745 | **.635** | **.636** | .743 | .744 | .743 | .744 | **.489** | **.487** |
|  |  |  | 30 | .746 | .746 | .746 | .746 | **.638** | **.637** | .743 | .743 | .743 | .743 | **.490** | **.490** |
|  |  |  | 50 | .744 | .744 | .744 | .744 | **.636** | **.636** | .744 | .744 | .744 | .744 | **.493** | **.493** |
|  |  |  | 80 | .743 | .743 | .743 | .743 | **.636** | **.636** | .744 | .744 | .744 | .744 | **.494** | **.495** |
|  |  | 200 | 10 | .746 | .746 | .745 | .746 | **.633** | **.634** | .745 | .745 | .745 | .745 | **.480** | **.480** |
|  |  |  | 20 | .746 | .746 | .746 | .746 | **.637** | **.637** | .746 | .746 | .746 | .746 | **.490** | **.490** |
|  |  |  | 30 | .745 | .745 | .745 | .745 | **.636** | **.636** | .746 | .747 | .747 | .747 | **.493** | **.493** |
|  |  |  | 50 | .745 | .745 | .745 | .746 | **.637** | **.637** | .746 | .745 | .745 | .746 | **.494** | **.494** |
|  |  |  | 80 | .745 | .745 | .745 | .745 | **.637** | **.638** | .746 | .746 | .746 | .746 | **.496** | **.496** |
| 3 | 1 | 50 | 10 | .245 | .243 | **.180** | .243 | **.182** | .245 | .245 | .246 | **.127** | .246 | **.127** | .245 |
|  |  |  | 20 | .246 | .246 | **.184** | .248 | **.183** | .248 | .245 | .245 | **.128** | .244 | **.129** | .245 |
|  |  |  | 30 | .247 | .246 | **.182** | .246 | **.183** | .247 | .245 | .245 | **.130** | .246 | **.130** | .246 |
|  |  |  | 50 | .247 | .246 | **.183** | .247 | **.183** | .247 | .245 | .245 | **.130** | .244 | **.130** | .244 |
|  |  |  | 80 | .247 | .247 | **.183** | .247 | **.183** | .247 | .245 | .245 | **.131** | .245 | **.131** | .245 |
|  |  | 100 | 10 | .246 | .247 | **.180** | .244 | **.181** | .247 | .248 | .247 | **.128** | .248 | **.128** | .249 |
|  |  |  | 20 | .245 | .245 | **.181** | .245 | **.181** | .245 | .246 | .246 | **.130** | .246 | **.129** | .246 |
|  |  |  | 30 | .246 | .245 | **.181** | .245 | **.181** | .246 | .246 | .246 | **.130** | .246 | **.131** | .246 |
|  |  |  | 50 | .246 | .247 | **.182** | .246 | **.183** | .247 | .247 | .247 | **.132** | .247 | **.132** | .247 |
|  |  |  | 80 | .247 | .247 | **.183** | .247 | **.183** | .247 | .246 | .246 | **.131** | .246 | **.131** | .246 |
|  |  | 200 | 10 | .248 | .248 | **.182** | .248 | **.182** | .247 | .246 | .246 | **.127** | .245 | **.126** | .246 |
|  |  |  | 20 | .248 | .248 | **.183** | .248 | **.183** | .247 | .247 | .248 | **.130** | .247 | **.130** | .247 |
|  |  |  | 30 | .247 | .248 | **.183** | .247 | **.183** | .247 | .247 | .246 | **.131** | .246 | **.130** | .246 |
|  |  |  | 50 | .246 | .246 | **.182** | .246 | **.182** | .246 | .246 | .246 | **.131** | .246 | **.131** | .246 |
|  |  |  | 80 | .246 | .246 | **.182** | .246 | **.183** | .247 | .247 | .247 | **.131** | .247 | **.132** | .247 |
|  | 2 | 50 | 10 | .558 | .556 | **.448** | .558 | **.449** | .557 | .560 | .560 | **.332** | .560 | **.332** | .560 |
|  |  |  | 20 | .558 | .559 | **.451** | .558 | **.451** | .558 | .563 | .563 | **.340** | .562 | **.340** | .563 |
|  |  |  | 30 | .563 | .564 | **.456** | .563 | **.456** | .562 | .564 | .564 | **.342** | .564 | **.342** | .564 |
|  |  |  | 50 | .561 | .561 | **.454** | .561 | **.454** | .561 | .562 | .562 | **.342** | .562 | **.342** | .562 |
|  |  |  | 80 | .561 | .562 | **.455** | .561 | **.455** | .562 | .562 | .562 | **.343** | .562 | **.344** | .562 |
|  |  | 100 | 10 | .565 | .565 | **.455** | .566 | **.454** | .566 | .566 | .567 | **.335** | .566 | **.337** | .566 |
|  |  |  | 20 | .564 | .564 | **.455** | .564 | **.456** | .564 | .565 | .565 | **.340** | .565 | **.341** | .565 |
|  |  |  | 30 | .564 | .564 | **.457** | .564 | **.457** | .565 | .565 | .565 | **.342** | .566 | **.343** | .565 |
|  |  |  | 50 | .565 | .565 | **.458** | .565 | **.457** | .565 | .565 | .565 | **.345** | .565 | **.344** | .565 |
|  |  |  | 80 | .563 | .564 | **.456** | .564 | **.456** | .564 | .564 | .564 | **.344** | .564 | **.345** | .564 |
|  |  | 200 | 10 | .567 | .567 | **.456** | .566 | **.455** | .567 | .566 | .566 | **.335** | .566 | **.335** | .566 |
|  |  |  | 20 | .567 | .567 | **.458** | .568 | **.459** | .568 | .565 | .565 | **.341** | .566 | **.341** | .566 |
|  |  |  | 30 | .566 | .566 | **.458** | .566 | **.458** | .566 | .566 | .566 | **.343** | .566 | **.343** | .566 |
|  |  |  | 50 | .567 | .567 | **.460** | .567 | **.460** | .567 | .566 | .566 | **.345** | .567 | **.346** | .567 |
|  |  |  | 80 | .565 | .565 | **.458** | .565 | **.457** | .565 | .565 | .565 | **.345** | .565 | **.345** | .565 |
|  | 3 | 50 | 10 | .740 | .741 | **.629** | .741 | **.629** | .742 | .742 | .741 | **.477** | .741 | **.477** | .741 |
|  |  |  | 20 | .742 | .742 | **.632** | .742 | **.632** | .742 | .741 | .741 | **.486** | .741 | **.486** | .741 |
|  |  |  | 30 | .741 | .742 | **.634** | .742 | **.634** | .742 | .742 | .742 | **.489** | .742 | **.490** | .742 |
|  |  |  | 50 | .742 | .741 | **.634** | .742 | **.634** | .742 | .741 | .741 | **.491** | .742 | **.491** | .741 |
|  |  |  | 80 | .741 | .741 | **.634** | .740 | **.633** | .741 | .743 | .743 | **.493** | .743 | **.494** | .743 |
|  |  | 100 | 10 | .745 | .745 | **.632** | .745 | **.633** | .745 | .742 | .742 | **.477** | .742 | **.478** | .742 |
|  |  |  | 20 | .744 | .744 | **.634** | .744 | **.635** | .744 | .745 | .745 | **.488** | .745 | **.489** | .744 |
|  |  |  | 30 | .743 | .743 | **.635** | .743 | **.634** | .743 | .745 | .745 | **.492** | .745 | **.492** | .745 |
|  |  |  | 50 | .744 | .744 | **.636** | .744 | **.636** | .744 | .744 | .744 | **.494** | .744 | **.494** | .744 |
|  |  |  | 80 | .745 | .745 | **.637** | .745 | **.637** | .745 | .744 | .743 | **.494** | .743 | **.494** | .744 |
|  |  | 200 | 10 | .746 | .746 | **.633** | .746 | **.634** | .746 | .747 | .747 | **.482** | .747 | **.482** | .747 |
|  |  |  | 20 | .745 | .745 | **.636** | .746 | **.636** | .746 | .747 | .746 | **.490** | .746 | **.491** | .746 |
|  |  |  | 30 | .746 | .746 | **.637** | .746 | **.638** | .746 | .745 | .745 | **.492** | .745 | **.492** | .745 |
|  |  |  | 50 | .747 | .747 | **.639** | .747 | **.639** | .746 | .745 | .745 | **.494** | .745 | **.494** | .745 |
|  |  |  | 80 | .747 | .747 | **.639** | .747 | **.639** | .747 | .746 | .746 | **.497** | .746 | **.497** | .746 |
| 4 | 1 | 50 | 10 | .245 | .247 | **.181** | **.182** | **.180** | **.181** | .246 | .246 | **.127** | **.127** | **.127** | **.129** |
|  |  |  | 20 | .247 | .248 | **.182** | **.182** | **.182** | **.182** | .243 | .242 | **.128** | **.128** | **.128** | **.128** |
|  |  |  | 30 | .246 | .245 | **.182** | **.181** | **.180** | **.181** | .245 | .245 | **.130** | **.130** | **.128** | **.129** |
|  |  |  | 50 | .247 | .247 | **.183** | **.183** | **.183** | **.184** | .245 | .245 | **.131** | **.131** | **.130** | **.132** |
|  |  |  | 80 | .247 | .247 | **.183** | **.183** | **.183** | **.183** | .244 | .244 | **.130** | **.131** | **.130** | **.130** |
|  |  | 100 | 10 | .248 | .246 | **.181** | **.181** | **.181** | **.181** | .247 | .248 | **.127** | **.128** | **.128** | **.128** |
|  |  |  | 20 | .247 | .246 | **.182** | **.182** | **.183** | **.183** | .245 | .244 | **.129** | **.129** | **.129** | **.129** |
|  |  |  | 30 | .246 | .246 | **.182** | **.182** | **.182** | **.182** | .246 | .246 | **.130** | **.130** | **.130** | **.130** |
|  |  |  | 50 | .246 | .246 | **.182** | **.183** | **.183** | **.183** | .248 | .248 | **.132** | **.132** | **.132** | **.132** |
|  |  |  | 80 | .247 | .247 | **.183** | **.183** | **.183** | **.183** | .246 | .246 | **.131** | **.131** | **.131** | **.131** |
|  |  | 200 | 10 | .247 | .247 | **.181** | **.181** | **.181** | **.181** | .248 | .247 | **.127** | **.127** | **.128** | **.128** |
|  |  |  | 20 | .247 | .247 | **.183** | **.182** | **.182** | **.183** | .248 | .247 | **.130** | **.130** | **.130** | **.130** |
|  |  |  | 30 | .247 | .247 | **.183** | **.182** | **.182** | **.182** | .248 | .248 | **.131** | **.131** | **.131** | **.131** |
|  |  |  | 50 | .246 | .246 | **.182** | **.182** | **.182** | **.182** | .247 | .247 | **.131** | **.131** | **.131** | **.131** |
|  |  |  | 80 | .247 | .247 | **.183** | **.183** | **.183** | **.183** | .246 | .246 | **.131** | **.131** | **.131** | **.131** |
|  | 2 | 50 | 10 | .564 | .564 | **.454** | **.453** | **.454** | **.454** | .563 | .562 | **.333** | **.333** | **.334** | **.331** |
|  |  |  | 20 | .563 | .563 | **.455** | **.454** | **.454** | **.454** | .563 | .563 | **.340** | **.340** | **.340** | **.341** |
|  |  |  | 30 | .564 | .564 | **.455** | **.456** | **.455** | **.455** | .564 | .564 | **.342** | **.342** | **.342** | **.343** |
|  |  |  | 50 | .559 | .559 | **.453** | **.452** | **.453** | **.453** | .563 | .563 | **.343** | **.343** | **.343** | **.343** |
|  |  |  | 80 | .561 | .560 | **.453** | **.454** | **.454** | **.454** | .563 | .563 | **.343** | **.344** | **.343** | **.343** |
|  |  | 100 | 10 | .565 | .565 | **.455** | **.455** | **.454** | **.456** | .563 | .563 | **.333** | **.334** | **.333** | **.333** |
|  |  |  | 20 | .562 | .563 | **.455** | **.454** | **.455** | **.454** | .565 | .565 | **.340** | **.341** | **.340** | **.341** |
|  |  |  | 30 | .567 | .568 | **.460** | **.460** | **.460** | **.459** | .565 | .565 | **.343** | **.343** | **.343** | **.343** |
|  |  |  | 50 | .566 | .566 | **.458** | **.458** | **.458** | **.458** | .565 | .565 | **.344** | **.344** | **.345** | **.344** |
|  |  |  | 80 | .565 | .565 | **.457** | **.457** | **.458** | **.457** | .564 | .563 | **.344** | **.343** | **.344** | **.344** |
|  |  | 200 | 10 | .566 | .566 | **.456** | **.456** | **.455** | **.455** | .567 | .567 | **.335** | **.335** | **.335** | **.336** |
|  |  |  | 20 | .565 | .566 | **.457** | **.456** | **.456** | **.457** | .566 | .566 | **.342** | **.341** | **.341** | **.341** |
|  |  |  | 30 | .567 | .567 | **.459** | **.459** | **.459** | **.458** | .565 | .565 | **.343** | **.343** | **.343** | **.343** |
|  |  |  | 50 | .567 | .567 | **.460** | **.459** | **.460** | **.460** | .567 | .567 | **.345** | **.345** | **.345** | **.346** |
|  |  |  | 80 | .565 | .565 | **.458** | **.458** | **.458** | **.458** | .567 | .567 | **.346** | **.346** | **.346** | **.346** |
|  | 3 | 50 | 10 | .741 | .742 | **.630** | **.629** | **.630** | **.630** | .741 | .741 | **.476** | **.477** | **.477** | **.477** |
|  |  |  | 20 | .741 | .740 | **.632** | **.630** | **.631** | **.631** | .742 | .742 | **.487** | **.487** | **.486** | **.486** |
|  |  |  | 30 | .739 | .739 | **.630** | **.631** | **.631** | **.631** | .742 | .741 | **.489** | **.489** | **.489** | **.489** |
|  |  |  | 50 | .741 | .741 | **.634** | **.634** | **.633** | **.633** | .740 | .741 | **.491** | **.490** | **.491** | **.490** |
|  |  |  | 80 | .741 | .741 | **.634** | **.634** | **.634** | **.634** | .740 | .740 | **.491** | **.492** | **.492** | **.492** |
|  |  | 100 | 10 | .743 | .744 | **.631** | **.631** | **.632** | **.632** | .745 | .744 | **.480** | **.480** | **.480** | **.480** |
|  |  |  | 20 | .743 | .744 | **.635** | **.634** | **.634** | **.635** | .744 | .744 | **.488** | **.488** | **.489** | **.489** |
|  |  |  | 30 | .744 | .744 | **.636** | **.635** | **.635** | **.635** | .744 | .744 | **.491** | **.492** | **.491** | **.491** |
|  |  |  | 50 | .744 | .744 | **.635** | **.636** | **.636** | **.636** | .745 | .745 | **.495** | **.495** | **.494** | **.495** |
|  |  |  | 80 | .743 | .743 | **.636** | **.636** | **.636** | **.636** | .745 | .745 | **.496** | **.495** | **.495** | **.495** |
|  |  | 200 | 10 | .746 | .746 | **.633** | **.633** | **.633** | **.633** | .747 | .747 | **.482** | **.481** | **.482** | **.482** |
|  |  |  | 20 | .746 | .746 | **.637** | **.637** | **.637** | **.637** | .746 | .746 | **.490** | **.490** | **.490** | **.491** |
|  |  |  | 30 | .746 | .747 | **.638** | **.638** | **.638** | **.638** | .746 | .746 | **.493** | **.493** | **.493** | **.493** |
|  |  |  | 50 | .746 | .746 | **.638** | **.638** | **.638** | **.638** | .746 | .746 | **.495** | **.495** | **.495** | **.495** |
|  |  |  | 80 | .745 | .745 | **.638** | **.638** | **.638** | **.638** | .745 | .745 | **.496** | **.495** | **.495** | **.495** |

# Results: bias condition none (Type I error)

Table A2

Fit indices’ means and standard deviations for varying ICCs (low., med. = medium, high), number of participants (n), number of re-tests (t) and for *bias condition none* with low/high bias. Please note that in this bias condition the varying strength of the bias was irrelevant. We nevertheless present both results to keep with our simulation design.

|  |  |  | Low | | | | | | | | High | | | | | | | | |
| --- | --- | --- | --- | --- | --- | --- | --- | --- | --- | --- | --- | --- | --- | --- | --- | --- | --- | --- | --- |
|  |  |  | CFI | | RMSEA | | SRMR-b | | SRMR-w | | CFI | | RMSEA | | | SRMR-b | | SRMR-w | |
| ICC | n | t | M | SD | M | SD | M | SD | M | SD | M | SD | | M | SD | M | SD | M | SD |
| low | 50 | 10 | 1 | .002 | .001 | .005 | .017 | .008 | .020 | .005 | 1 | .001 | | .001 | .005 | .017 | .008 | .020 | .005 |
|  |  | 20 | 1 | .001 | .001 | .003 | .009 | .004 | .014 | .003 | 1 | .001 | | .001 | .004 | .008 | .004 | .014 | .003 |
|  |  | 30 | 1 | .001 | .001 | .003 | .006 | .002 | .011 | .003 | 1 | .001 | | .001 | .003 | .005 | .002 | .011 | .003 |
|  |  | 50 | 1 | 0 | 0 | .002 | .003 | .001 | .009 | .002 | 1 | 0 | | 0 | .002 | .003 | .001 | .009 | .002 |
|  |  | 80 | 1 | 0 | 0 | .002 | .002 | .001 | .007 | .002 | 1 | 0 | | 0 | .001 | .002 | .001 | .007 | .002 |
|  | 100 | 10 | 1 | .001 | .001 | .004 | .012 | .004 | .014 | .003 | 1 | .001 | | .001 | .004 | .012 | .005 | .014 | .003 |
|  |  | 20 | 1 | 0 | .001 | .002 | .006 | .002 | .010 | .002 | 1 | 0 | | .001 | .003 | .006 | .002 | .010 | .002 |
|  |  | 30 | 1 | 0 | 0 | .002 | .004 | .001 | .008 | .002 | 1 | 0 | | 0 | .002 | .004 | .001 | .008 | .002 |
|  |  | 50 | 1 | 0 | 0 | .001 | .002 | .001 | .006 | .001 | 1 | 0 | | 0 | .001 | .002 | .001 | .006 | .001 |
|  |  | 80 | 1 | 0 | 0 | .001 | .001 | .001 | .005 | .001 | 1 | 0 | | 0 | .001 | .001 | .001 | .005 | .001 |
|  | 200 | 10 | 1 | 0 | .001 | .003 | .008 | .003 | .010 | .002 | 1 | 0 | | .001 | .003 | .008 | .003 | .010 | .002 |
|  |  | 20 | 1 | 0 | 0 | .002 | .004 | .001 | .007 | .002 | 1 | 0 | | .001 | .002 | .004 | .001 | .007 | .002 |
|  |  | 30 | 1 | 0 | 0 | .002 | .003 | .001 | .006 | .001 | 1 | 0 | | 0 | .002 | .003 | .001 | .006 | .001 |
|  |  | 50 | 1 | 0 | 0 | .001 | .002 | .001 | .004 | .001 | 1 | 0 | | 0 | .001 | .002 | .001 | .004 | .001 |
|  |  | 80 | 1 | 0 | 0 | .001 | .001 | 0 | .003 | .001 | 1 | 0 | | 0 | .001 | .001 | 0 | .003 | .001 |
| med. | 50 | 10 | 1 | .001 | .001 | .004 | .004 | .002 | .020 | .005 | 1 | .001 | | .001 | .005 | .004 | .002 | .020 | .005 |
|  |  | 20 | 1 | .001 | .001 | .003 | .002 | .001 | .014 | .003 | 1 | .001 | | .001 | .003 | .002 | .001 | .014 | .003 |
|  |  | 30 | 1 | 0 | 0 | .002 | .001 | .001 | .011 | .003 | 1 | 0 | | .001 | .003 | .001 | .001 | .011 | .003 |
|  |  | 50 | 1 | 0 | 0 | .002 | .001 | 0 | .009 | .002 | 1 | 0 | | 0 | .002 | .001 | 0 | .009 | .002 |
|  |  | 80 | 1 | 0 | 0 | .001 | .001 | 0 | .007 | .002 | 1 | 0 | | 0 | .002 | .001 | 0 | .007 | .002 |
|  | 100 | 10 | 1 | .001 | .001 | .004 | .003 | .001 | .014 | .003 | 1 | .001 | | .001 | .004 | .003 | .001 | .014 | .003 |
|  |  | 20 | 1 | 0 | .001 | .003 | .001 | .001 | .010 | .002 | 1 | 0 | | .001 | .003 | .001 | .001 | .010 | .002 |
|  |  | 30 | 1 | 0 | 0 | .002 | .001 | 0 | .008 | .002 | 1 | 0 | | 0 | .002 | .001 | 0 | .008 | .002 |
|  |  | 50 | 1 | 0 | 0 | .002 | .001 | 0 | .006 | .001 | 1 | 0 | | 0 | .002 | .001 | 0 | .006 | .002 |
|  |  | 80 | 1 | 0 | 0 | .001 | 0 | 0 | .005 | .001 | 1 | 0 | | 0 | .001 | 0 | 0 | .005 | .001 |
|  | 200 | 10 | 1 | 0 | .001 | .003 | .002 | .001 | .010 | .002 | 1 | 0 | | .001 | .003 | .002 | .001 | .010 | .002 |
|  |  | 20 | 1 | 0 | 0 | .002 | .001 | 0 | .007 | .002 | 1 | 0 | | 0 | .002 | .001 | 0 | .007 | .002 |
|  |  | 30 | 1 | 0 | 0 | .002 | .001 | 0 | .006 | .001 | 1 | 0 | | 0 | .001 | .001 | 0 | .006 | .001 |
|  |  | 50 | 1 | 0 | 0 | .001 | 0 | 0 | .004 | .001 | 1 | 0 | | 0 | .001 | 0 | 0 | .004 | .001 |
|  |  | 80 | 1 | 0 | 0 | .001 | 0 | 0 | .003 | .001 | 1 | 0 | | 0 | .001 | 0 | 0 | .003 | .001 |
| high | 50 | 10 | 1 | .001 | .001 | .004 | .002 | .001 | .020 | .004 | 1 | .001 | | .001 | .004 | .002 | .001 | .020 | .004 |
|  |  | 20 | 1 | .001 | .001 | .003 | .001 | 0 | .014 | .003 | 1 | 0 | | .001 | .003 | .001 | 0 | .014 | .003 |
|  |  | 30 | 1 | 0 | 0 | .002 | .001 | 0 | .011 | .003 | 1 | 0 | | 0 | .002 | .001 | 0 | .011 | .003 |
|  |  | 50 | 1 | 0 | 0 | .002 | 0 | 0 | .009 | .002 | 1 | 0 | | 0 | .002 | 0 | 0 | .009 | .002 |
|  |  | 80 | 1 | 0 | 0 | .002 | 0 | 0 | .007 | .002 | 1 | 0 | | 0 | .002 | 0 | 0 | .007 | .002 |
|  | 100 | 10 | 1 | 0 | .001 | .003 | .001 | .001 | .014 | .003 | 1 | 0 | | .001 | .003 | .001 | .001 | .014 | .003 |
|  |  | 20 | 1 | 0 | .001 | .003 | .001 | 0 | .010 | .002 | 1 | 0 | | .001 | .002 | .001 | 0 | .010 | .002 |
|  |  | 30 | 1 | 0 | 0 | .002 | 0 | 0 | .008 | .002 | 1 | 0 | | 0 | .002 | 0 | 0 | .008 | .002 |
|  |  | 50 | 1 | 0 | 0 | .002 | 0 | 0 | .006 | .001 | 1 | 0 | | 0 | .001 | 0 | 0 | .006 | .001 |
|  |  | 80 | 1 | 0 | 0 | .001 | 0 | 0 | .005 | .001 | 1 | 0 | | 0 | .001 | 0 | 0 | .005 | .001 |
|  | 200 | 10 | 1 | 0 | .001 | .003 | .001 | 0 | .010 | .002 | 1 | 0 | | .001 | .003 | .001 | 0 | .010 | .002 |
|  |  | 20 | 1 | 0 | .001 | .002 | 0 | 0 | .007 | .002 | 1 | 0 | | 0 | .002 | 0 | 0 | .007 | .002 |
|  |  | 30 | 1 | 0 | 0 | .001 | 0 | 0 | .006 | .001 | 1 | 0 | | 0 | .002 | 0 | 0 | .006 | .001 |
|  |  | 50 | 1 | 0 | 0 | .001 | 0 | 0 | .004 | .001 | 1 | 0 | | 0 | .001 | 0 | 0 | .004 | .001 |
|  |  | 80 | 1 | 0 | 0 | .001 | 0 | 0 | .003 | .001 | 1 | 0 | | 0 | .001 | 0 | 0 | .003 | .001 |

Table A3

Percentages of fit indices above/below their cut-off according to common SEM cut-offs for varying ICCs (low., med. = medium, high), number of participants (n), number of re-tests (t) and for *bias condition none* with low/high bias. For p-values, the absolute number of how often p < .05 in the 1000 simulations is given. Please note that in this bias condition the varying strength of the bias was irrelevant. We nevertheless present both results to keep with our simulation design.

|  |  |  | Low |  |  |  |  |  |  |  |  |  |  | High |  |  |  |  |  |  |  |  |  |  |
| --- | --- | --- | --- | --- | --- | --- | --- | --- | --- | --- | --- | --- | --- | --- | --- | --- | --- | --- | --- | --- | --- | --- | --- | --- |
|  |  |  | p | CFI | | | RMSEA | | | SRMR-b | | SRMR-within | | p | CFI | | | RMSEA | | | SRMR-b | | SRMR-within | |
| ICC | n | t | <.05 | ≥.99 | ≥.95 | ≥.90 | <.06 | <.08 | <.10 | <.08 | <.11 | <.08 | <.11 | <.05 | ≥.99 | ≥.95 | ≥.90 | <.06 | <.08 | <.10 | <.08 | <.11 | <.08 | <.11 |
| low | 50 | 10 | 4 | .994 | 1 | 1 | 1 | 1 | 1 | .999 | 1 | 1 | 1 | 1 | .994 | 1 | 1 | 1 | 1 | 1 | 1 | 1 | 1 | 1 |
|  |  | 20 | 0 | 1 | 1 | 1 | 1 | 1 | 1 | 1 | 1 | 1 | 1 | 4 | .999 | 1 | 1 | 1 | 1 | 1 | 1 | 1 | 1 | 1 |
|  |  | 30 | 2 | .999 | 1 | 1 | 1 | 1 | 1 | 1 | 1 | 1 | 1 | 1 | 1 | 1 | 1 | 1 | 1 | 1 | 1 | 1 | 1 | 1 |
|  |  | 50 | 4 | 1 | 1 | 1 | 1 | 1 | 1 | 1 | 1 | 1 | 1 | 4 | 1 | 1 | 1 | 1 | 1 | 1 | 1 | 1 | 1 | 1 |
|  |  | 80 | 3 | 1 | 1 | 1 | 1 | 1 | 1 | 1 | 1 | 1 | 1 | 1 | 1 | 1 | 1 | 1 | 1 | 1 | 1 | 1 | 1 | 1 |
|  | 100 | 10 | 2 | 1 | 1 | 1 | 1 | 1 | 1 | 1 | 1 | 1 | 1 | 2 | 1 | 1 | 1 | 1 | 1 | 1 | 1 | 1 | 1 | 1 |
|  |  | 20 | 4 | 1 | 1 | 1 | 1 | 1 | 1 | 1 | 1 | 1 | 1 | 3 | 1 | 1 | 1 | 1 | 1 | 1 | 1 | 1 | 1 | 1 |
|  |  | 30 | 4 | 1 | 1 | 1 | 1 | 1 | 1 | 1 | 1 | 1 | 1 | 2 | 1 | 1 | 1 | 1 | 1 | 1 | 1 | 1 | 1 | 1 |
|  |  | 50 | 0 | 1 | 1 | 1 | 1 | 1 | 1 | 1 | 1 | 1 | 1 | 1 | 1 | 1 | 1 | 1 | 1 | 1 | 1 | 1 | 1 | 1 |
|  |  | 80 | 1 | 1 | 1 | 1 | 1 | 1 | 1 | 1 | 1 | 1 | 1 | 1 | 1 | 1 | 1 | 1 | 1 | 1 | 1 | 1 | 1 | 1 |
|  | 200 | 10 | 1 | 1 | 1 | 1 | 1 | 1 | 1 | 1 | 1 | 1 | 1 | 1 | 1 | 1 | 1 | 1 | 1 | 1 | 1 | 1 | 1 | 1 |
|  |  | 20 | 1 | 1 | 1 | 1 | 1 | 1 | 1 | 1 | 1 | 1 | 1 | 6 | 1 | 1 | 1 | 1 | 1 | 1 | 1 | 1 | 1 | 1 |
|  |  | 30 | 4 | 1 | 1 | 1 | 1 | 1 | 1 | 1 | 1 | 1 | 1 | 1 | 1 | 1 | 1 | 1 | 1 | 1 | 1 | 1 | 1 | 1 |
|  |  | 50 | 3 | 1 | 1 | 1 | 1 | 1 | 1 | 1 | 1 | 1 | 1 | 3 | 1 | 1 | 1 | 1 | 1 | 1 | 1 | 1 | 1 | 1 |
|  |  | 80 | 4 | 1 | 1 | 1 | 1 | 1 | 1 | 1 | 1 | 1 | 1 | 5 | 1 | 1 | 1 | 1 | 1 | 1 | 1 | 1 | 1 | 1 |
| med. | 50 | 10 | 2 | .998 | 1 | 1 | 1 | 1 | 1 | 1 | 1 | 1 | 1 | 2 | .998 | 1 | 1 | 1 | 1 | 1 | 1 | 1 | 1 | 1 |
|  |  | 20 | 3 | 1 | 1 | 1 | 1 | 1 | 1 | 1 | 1 | 1 | 1 | 3 | 1 | 1 | 1 | 1 | 1 | 1 | 1 | 1 | 1 | 1 |
|  |  | 30 | 1 | 1 | 1 | 1 | 1 | 1 | 1 | 1 | 1 | 1 | 1 | 2 | 1 | 1 | 1 | 1 | 1 | 1 | 1 | 1 | 1 | 1 |
|  |  | 50 | 3 | 1 | 1 | 1 | 1 | 1 | 1 | 1 | 1 | 1 | 1 | 3 | 1 | 1 | 1 | 1 | 1 | 1 | 1 | 1 | 1 | 1 |
|  |  | 80 | 1 | 1 | 1 | 1 | 1 | 1 | 1 | 1 | 1 | 1 | 1 | 2 | 1 | 1 | 1 | 1 | 1 | 1 | 1 | 1 | 1 | 1 |
|  | 100 | 10 | 3 | 1 | 1 | 1 | 1 | 1 | 1 | 1 | 1 | 1 | 1 | 3 | 1 | 1 | 1 | 1 | 1 | 1 | 1 | 1 | 1 | 1 |
|  |  | 20 | 3 | 1 | 1 | 1 | 1 | 1 | 1 | 1 | 1 | 1 | 1 | 4 | 1 | 1 | 1 | 1 | 1 | 1 | 1 | 1 | 1 | 1 |
|  |  | 30 | 3 | 1 | 1 | 1 | 1 | 1 | 1 | 1 | 1 | 1 | 1 | 1 | 1 | 1 | 1 | 1 | 1 | 1 | 1 | 1 | 1 | 1 |
|  |  | 50 | 2 | 1 | 1 | 1 | 1 | 1 | 1 | 1 | 1 | 1 | 1 | 1 | 1 | 1 | 1 | 1 | 1 | 1 | 1 | 1 | 1 | 1 |
|  |  | 80 | 4 | 1 | 1 | 1 | 1 | 1 | 1 | 1 | 1 | 1 | 1 | 2 | 1 | 1 | 1 | 1 | 1 | 1 | 1 | 1 | 1 | 1 |
|  | 200 | 10 | 0 | 1 | 1 | 1 | 1 | 1 | 1 | 1 | 1 | 1 | 1 | 5 | 1 | 1 | 1 | 1 | 1 | 1 | 1 | 1 | 1 | 1 |
|  |  | 20 | 1 | 1 | 1 | 1 | 1 | 1 | 1 | 1 | 1 | 1 | 1 | 1 | 1 | 1 | 1 | 1 | 1 | 1 | 1 | 1 | 1 | 1 |
|  |  | 30 | 2 | 1 | 1 | 1 | 1 | 1 | 1 | 1 | 1 | 1 | 1 | 2 | 1 | 1 | 1 | 1 | 1 | 1 | 1 | 1 | 1 | 1 |
|  |  | 50 | 0 | 1 | 1 | 1 | 1 | 1 | 1 | 1 | 1 | 1 | 1 | 3 | 1 | 1 | 1 | 1 | 1 | 1 | 1 | 1 | 1 | 1 |
|  |  | 80 | 3 | 1 | 1 | 1 | 1 | 1 | 1 | 1 | 1 | 1 | 1 | 3 | 1 | 1 | 1 | 1 | 1 | 1 | 1 | 1 | 1 | 1 |
| high | 50 | 10 | 1 | 1 | 1 | 1 | 1 | 1 | 1 | 1 | 1 | 1 | 1 | 0 | 1 | 1 | 1 | 1 | 1 | 1 | 1 | 1 | 1 | 1 |
|  |  | 20 | 4 | .999 | 1 | 1 | 1 | 1 | 1 | 1 | 1 | 1 | 1 | 0 | 1 | 1 | 1 | 1 | 1 | 1 | 1 | 1 | 1 | 1 |
|  |  | 30 | 1 | 1 | 1 | 1 | 1 | 1 | 1 | 1 | 1 | 1 | 1 | 2 | 1 | 1 | 1 | 1 | 1 | 1 | 1 | 1 | 1 | 1 |
|  |  | 50 | 0 | 1 | 1 | 1 | 1 | 1 | 1 | 1 | 1 | 1 | 1 | 4 | 1 | 1 | 1 | 1 | 1 | 1 | 1 | 1 | 1 | 1 |
|  |  | 80 | 3 | 1 | 1 | 1 | 1 | 1 | 1 | 1 | 1 | 1 | 1 | 3 | 1 | 1 | 1 | 1 | 1 | 1 | 1 | 1 | 1 | 1 |
|  | 100 | 10 | 0 | 1 | 1 | 1 | 1 | 1 | 1 | 1 | 1 | 1 | 1 | 1 | 1 | 1 | 1 | 1 | 1 | 1 | 1 | 1 | 1 | 1 |
|  |  | 20 | 2 | 1 | 1 | 1 | 1 | 1 | 1 | 1 | 1 | 1 | 1 | 2 | 1 | 1 | 1 | 1 | 1 | 1 | 1 | 1 | 1 | 1 |
|  |  | 30 | 5 | 1 | 1 | 1 | 1 | 1 | 1 | 1 | 1 | 1 | 1 | 2 | 1 | 1 | 1 | 1 | 1 | 1 | 1 | 1 | 1 | 1 |
|  |  | 50 | 5 | 1 | 1 | 1 | 1 | 1 | 1 | 1 | 1 | 1 | 1 | 0 | 1 | 1 | 1 | 1 | 1 | 1 | 1 | 1 | 1 | 1 |
|  |  | 80 | 0 | 1 | 1 | 1 | 1 | 1 | 1 | 1 | 1 | 1 | 1 | 0 | 1 | 1 | 1 | 1 | 1 | 1 | 1 | 1 | 1 | 1 |
|  | 200 | 10 | 4 | 1 | 1 | 1 | 1 | 1 | 1 | 1 | 1 | 1 | 1 | 2 | 1 | 1 | 1 | 1 | 1 | 1 | 1 | 1 | 1 | 1 |
|  |  | 20 | 3 | 1 | 1 | 1 | 1 | 1 | 1 | 1 | 1 | 1 | 1 | 3 | 1 | 1 | 1 | 1 | 1 | 1 | 1 | 1 | 1 | 1 |
|  |  | 30 | 1 | 1 | 1 | 1 | 1 | 1 | 1 | 1 | 1 | 1 | 1 | 3 | 1 | 1 | 1 | 1 | 1 | 1 | 1 | 1 | 1 | 1 |
|  |  | 50 | 0 | 1 | 1 | 1 | 1 | 1 | 1 | 1 | 1 | 1 | 1 | 4 | 1 | 1 | 1 | 1 | 1 | 1 | 1 | 1 | 1 | 1 |
|  |  | 80 | 2 | 1 | 1 | 1 | 1 | 1 | 1 | 1 | 1 | 1 | 1 | 4 | 1 | 1 | 1 | 1 | 1 | 1 | 1 | 1 | 1 | 1 |

# Results: bias condition 1

Table A4

Fit indices’ means and standard deviations for varying ICCs (low., med. = medium, high), number of participants (n), number of re-tests (t) and for *bias condition 1* with low/high bias.

|  |  |  | Low | | | | | | | | High | | | | | | | | |
| --- | --- | --- | --- | --- | --- | --- | --- | --- | --- | --- | --- | --- | --- | --- | --- | --- | --- | --- | --- |
|  |  |  | CFI | | RMSEA | | SRMR-b | | SRMR-w | | CFI | | RMSEA | | | SRMR-b | | SRMR-w | |
| ICC | n | t | M | SD | M | SD | M | SD | M | SD | M | SD | | M | SD | M | SD | M | SD |
| low | 50 | 10 | 1 | .001 | .001 | .004 | .019 | .009 | .020 | .004 | 1 | .001 | | .001 | .004 | .022 | .010 | .021 | .005 |
|  |  | 20 | 1 | .001 | .001 | .004 | .009 | .004 | .014 | .003 | 1 | .001 | | 0 | .003 | .011 | .005 | .014 | .003 |
|  |  | 30 | 1 | .001 | .001 | .003 | .006 | .003 | .012 | .003 | 1 | 0 | | .001 | .002 | .007 | .003 | .012 | .003 |
|  |  | 50 | 1 | 0 | 0 | .002 | .004 | .002 | .009 | .002 | 1 | 0 | | 0 | .002 | .004 | .002 | .009 | .002 |
|  |  | 80 | 1 | 0 | 0 | .002 | .002 | .001 | .007 | .002 | 1 | 0 | | 0 | .001 | .003 | .001 | .007 | .002 |
|  | 100 | 10 | 1 | .001 | .001 | .004 | .013 | .005 | .015 | .003 | 1 | .001 | | .001 | .003 | .015 | .006 | .015 | .003 |
|  |  | 20 | 1 | 0 | .001 | .002 | .006 | .003 | .010 | .002 | 1 | 0 | | 0 | .002 | .007 | .003 | .010 | .002 |
|  |  | 30 | 1 | 0 | 0 | .002 | .004 | .002 | .008 | .002 | 1 | 0 | | 0 | .002 | .005 | .002 | .008 | .002 |
|  |  | 50 | 1 | 0 | 0 | .002 | .003 | .001 | .006 | .001 | 1 | 0 | | 0 | .001 | .003 | .001 | .006 | .001 |
|  |  | 80 | 1 | 0 | 0 | .001 | .002 | .001 | .005 | .001 | 1 | 0 | | 0 | .001 | .002 | .001 | .005 | .001 |
|  | 200 | 10 | 1 | 0 | .001 | .002 | .009 | .003 | .011 | .002 | 1 | 0 | | 0 | .002 | .010 | .004 | .010 | .002 |
|  |  | 20 | 1 | 0 | 0 | .002 | .005 | .002 | .007 | .002 | 1 | 0 | | 0 | .002 | .005 | .002 | .007 | .002 |
|  |  | 30 | 1 | 0 | 0 | .001 | .003 | .001 | .006 | .001 | 1 | 0 | | 0 | .002 | .003 | .001 | .006 | .001 |
|  |  | 50 | 1 | 0 | 0 | .001 | .002 | .001 | .004 | .001 | 1 | 0 | | 0 | .001 | .002 | .001 | .005 | .001 |
|  |  | 80 | 1 | 0 | 0 | .001 | .001 | 0 | .003 | .001 | 1 | 0 | | 0 | .001 | .001 | .001 | .004 | .001 |
| med. | 50 | 10 | 1 | .001 | .001 | .004 | .005 | .002 | .021 | .005 | 1 | .001 | | .001 | .004 | .005 | .003 | .022 | .005 |
|  |  | 20 | 1 | .001 | .001 | .003 | .002 | .001 | .014 | .003 | 1 | 0 | | 0 | .002 | .002 | .001 | .015 | .003 |
|  |  | 30 | 1 | 0 | 0 | .002 | .001 | .001 | .012 | .003 | 1 | 0 | | 0 | .002 | .002 | .001 | .012 | .003 |
|  |  | 50 | 1 | 0 | 0 | .002 | .001 | 0 | .009 | .002 | 1 | 0 | | 0 | .002 | .001 | .001 | .009 | .002 |
|  |  | 80 | 1 | 0 | 0 | .001 | .001 | 0 | .007 | .002 | 1 | 0 | | 0 | .001 | .001 | 0 | .007 | .002 |
|  | 100 | 10 | 1 | .001 | .001 | .003 | .003 | .001 | .015 | .003 | 1 | 0 | | .001 | .003 | .003 | .002 | .015 | .003 |
|  |  | 20 | 1 | 0 | 0 | .002 | .002 | .001 | .010 | .002 | 1 | 0 | | 0 | .002 | .002 | .001 | .010 | .002 |
|  |  | 30 | 1 | 0 | 0 | .002 | .001 | 0 | .008 | .002 | 1 | 0 | | 0 | .002 | .001 | .001 | .008 | .002 |
|  |  | 50 | 1 | 0 | 0 | .001 | .001 | 0 | .006 | .002 | 1 | 0 | | 0 | .001 | .001 | 0 | .006 | .002 |
|  |  | 80 | 1 | 0 | 0 | .001 | 0 | 0 | .005 | .001 | 1 | 0 | | 0 | .001 | 0 | 0 | .005 | .001 |
|  | 200 | 10 | 1 | 0 | 0 | .002 | .002 | .001 | .010 | .002 | 1 | 0 | | 0 | .001 | .002 | .001 | .011 | .002 |
|  |  | 20 | 1 | 0 | 0 | .002 | .001 | 0 | .007 | .002 | 1 | 0 | | 0 | .001 | .001 | 0 | .007 | .002 |
|  |  | 30 | 1 | 0 | 0 | .001 | .001 | 0 | .006 | .001 | 1 | 0 | | 0 | .001 | .001 | 0 | .006 | .001 |
|  |  | 50 | 1 | 0 | 0 | .001 | 0 | 0 | .005 | .001 | 1 | 0 | | 0 | .001 | 0 | 0 | .005 | .001 |
|  |  | 80 | 1 | 0 | 0 | .001 | 0 | 0 | .004 | .001 | 1 | 0 | | 0 | .001 | 0 | 0 | .004 | .001 |
| high | 50 | 10 | 1 | .001 | .001 | .004 | .002 | .001 | .021 | .005 | 1 | 0 | | 0 | .002 | .002 | .001 | .022 | .005 |
|  |  | 20 | 1 | 0 | .001 | .003 | .001 | 0 | .015 | .003 | 1 | 0 | | 0 | .002 | .001 | .001 | .015 | .003 |
|  |  | 30 | 1 | 0 | 0 | .002 | .001 | 0 | .012 | .003 | 1 | 0 | | 0 | .001 | .001 | 0 | .012 | .003 |
|  |  | 50 | 1 | 0 | 0 | .002 | 0 | 0 | .009 | .002 | 1 | 0 | | 0 | .001 | 0 | 0 | .009 | .002 |
|  |  | 80 | 1 | 0 | 0 | .001 | 0 | 0 | .007 | .002 | 1 | 0 | | 0 | .001 | 0 | 0 | .007 | .002 |
|  | 100 | 10 | 1 | 0 | 0 | .002 | .001 | .001 | .015 | .003 | 1 | 0 | | 0 | .001 | .001 | .001 | .015 | .003 |
|  |  | 20 | 1 | 0 | 0 | .002 | .001 | 0 | .010 | .002 | 1 | 0 | | 0 | .001 | .001 | 0 | .011 | .003 |
|  |  | 30 | 1 | 0 | 0 | .001 | 0 | 0 | .008 | .002 | 1 | 0 | | 0 | .001 | 0 | 0 | .008 | .002 |
|  |  | 50 | 1 | 0 | 0 | .001 | 0 | 0 | .006 | .001 | 1 | 0 | | 0 | 0 | 0 | 0 | .007 | .002 |
|  |  | 80 | 1 | 0 | 0 | .001 | 0 | 0 | .005 | .001 | 1 | 0 | | 0 | 0 | 0 | 0 | .005 | .001 |
|  | 200 | 10 | 1 | 0 | 0 | .002 | .001 | 0 | .011 | .002 | 1 | 0 | | 0 | 0 | .001 | 0 | .011 | .002 |
|  |  | 20 | 1 | 0 | 0 | .001 | 0 | 0 | .007 | .002 | 1 | 0 | | 0 | .001 | 0 | 0 | .007 | .002 |
|  |  | 30 | 1 | 0 | 0 | .001 | 0 | 0 | .006 | .001 | 1 | 0 | | 0 | 0 | 0 | 0 | .006 | .001 |
|  |  | 50 | 1 | 0 | 0 | .001 | 0 | 0 | .004 | .001 | 1 | 0 | | 0 | 0 | 0 | 0 | .005 | .001 |
|  |  | 80 | 1 | 0 | 0 | .001 | 0 | 0 | .004 | .001 | 1 | 0 | | 0 | 0 | 0 | 0 | .004 | .001 |

Table A5

Percentages of fit indices above/below their cut-off according to common SEM cut-offs for varying ICCs (low., med. = medium, high), number of participants (n), number of re-tests (t) and for *bias condition 1* with low/high bias. For p-values, the absolute number of how often p < .05 in the 1000 simulations is given.

|  |  |  | Low |  |  |  |  |  |  |  |  |  |  | High |  |  |  |  |  |  |  |  |  |  |
| --- | --- | --- | --- | --- | --- | --- | --- | --- | --- | --- | --- | --- | --- | --- | --- | --- | --- | --- | --- | --- | --- | --- | --- | --- |
|  |  |  | p | CFI | | | RMSEA | | | SRMR-b | | SRMR-within | | p | CFI | | | RMSEA | | | SRMR-b | | SRMR-within | |
| ICC | n | t | <.05 | ≥.99 | ≥.95 | ≥.90 | <.06 | <.08 | <.10 | <.08 | <.11 | <.08 | <.11 | <.05 | ≥.99 | ≥.95 | ≥.90 | <.06 | <.08 | <.10 | <.08 | <.11 | <.08 | <.11 |
| low | 50 | 10 | 0 | .999 | 1 | 1 | 1 | 1 | 1 | 1 | 1 | 1 | 1 | 2 | .995 | 1 | 1 | 1 | 1 | 1 | 1 | 1 | 1 | 1 |
|  |  | 20 | 3 | .999 | 1 | 1 | 1 | 1 | 1 | 1 | 1 | 1 | 1 | 1 | 1 | 1 | 1 | 1 | 1 | 1 | 1 | 1 | 1 | 1 |
|  |  | 30 | 1 | 1 | 1 | 1 | 1 | 1 | 1 | 1 | 1 | 1 | 1 | 0 | 1 | 1 | 1 | 1 | 1 | 1 | 1 | 1 | 1 | 1 |
|  |  | 50 | 0 | 1 | 1 | 1 | 1 | 1 | 1 | 1 | 1 | 1 | 1 | 1 | 1 | 1 | 1 | 1 | 1 | 1 | 1 | 1 | 1 | 1 |
|  |  | 80 | 2 | 1 | 1 | 1 | 1 | 1 | 1 | 1 | 1 | 1 | 1 | 0 | 1 | 1 | 1 | 1 | 1 | 1 | 1 | 1 | 1 | 1 |
|  | 100 | 10 | 3 | .999 | 1 | 1 | 1 | 1 | 1 | 1 | 1 | 1 | 1 | 3 | 1 | 1 | 1 | 1 | 1 | 1 | 1 | 1 | 1 | 1 |
|  |  | 20 | 1 | 1 | 1 | 1 | 1 | 1 | 1 | 1 | 1 | 1 | 1 | 2 | 1 | 1 | 1 | 1 | 1 | 1 | 1 | 1 | 1 | 1 |
|  |  | 30 | 0 | 1 | 1 | 1 | 1 | 1 | 1 | 1 | 1 | 1 | 1 | 2 | 1 | 1 | 1 | 1 | 1 | 1 | 1 | 1 | 1 | 1 |
|  |  | 50 | 4 | 1 | 1 | 1 | 1 | 1 | 1 | 1 | 1 | 1 | 1 | 2 | 1 | 1 | 1 | 1 | 1 | 1 | 1 | 1 | 1 | 1 |
|  |  | 80 | 3 | 1 | 1 | 1 | 1 | 1 | 1 | 1 | 1 | 1 | 1 | 2 | 1 | 1 | 1 | 1 | 1 | 1 | 1 | 1 | 1 | 1 |
|  | 200 | 10 | 2 | 1 | 1 | 1 | 1 | 1 | 1 | 1 | 1 | 1 | 1 | 4 | 1 | 1 | 1 | 1 | 1 | 1 | 1 | 1 | 1 | 1 |
|  |  | 20 | 0 | 1 | 1 | 1 | 1 | 1 | 1 | 1 | 1 | 1 | 1 | 3 | 1 | 1 | 1 | 1 | 1 | 1 | 1 | 1 | 1 | 1 |
|  |  | 30 | 2 | 1 | 1 | 1 | 1 | 1 | 1 | 1 | 1 | 1 | 1 | 4 | 1 | 1 | 1 | 1 | 1 | 1 | 1 | 1 | 1 | 1 |
|  |  | 50 | 2 | 1 | 1 | 1 | 1 | 1 | 1 | 1 | 1 | 1 | 1 | 2 | 1 | 1 | 1 | 1 | 1 | 1 | 1 | 1 | 1 | 1 |
|  |  | 80 | 3 | 1 | 1 | 1 | 1 | 1 | 1 | 1 | 1 | 1 | 1 | 0 | 1 | 1 | 1 | 1 | 1 | 1 | 1 | 1 | 1 | 1 |
| med. | 50 | 10 | 2 | .998 | 1 | 1 | 1 | 1 | 1 | 1 | 1 | 1 | 1 | 2 | .998 | 1 | 1 | 1 | 1 | 1 | 1 | 1 | 1 | 1 |
|  |  | 20 | 2 | 1 | 1 | 1 | 1 | 1 | 1 | 1 | 1 | 1 | 1 | 0 | 1 | 1 | 1 | 1 | 1 | 1 | 1 | 1 | 1 | 1 |
|  |  | 30 | 2 | 1 | 1 | 1 | 1 | 1 | 1 | 1 | 1 | 1 | 1 | 0 | 1 | 1 | 1 | 1 | 1 | 1 | 1 | 1 | 1 | 1 |
|  |  | 50 | 2 | 1 | 1 | 1 | 1 | 1 | 1 | 1 | 1 | 1 | 1 | 0 | 1 | 1 | 1 | 1 | 1 | 1 | 1 | 1 | 1 | 1 |
|  |  | 80 | 0 | 1 | 1 | 1 | 1 | 1 | 1 | 1 | 1 | 1 | 1 | 1 | 1 | 1 | 1 | 1 | 1 | 1 | 1 | 1 | 1 | 1 |
|  | 100 | 10 | 1 | .999 | 1 | 1 | 1 | 1 | 1 | 1 | 1 | 1 | 1 | 0 | 1 | 1 | 1 | 1 | 1 | 1 | 1 | 1 | 1 | 1 |
|  |  | 20 | 2 | 1 | 1 | 1 | 1 | 1 | 1 | 1 | 1 | 1 | 1 | 1 | 1 | 1 | 1 | 1 | 1 | 1 | 1 | 1 | 1 | 1 |
|  |  | 30 | 3 | 1 | 1 | 1 | 1 | 1 | 1 | 1 | 1 | 1 | 1 | 2 | 1 | 1 | 1 | 1 | 1 | 1 | 1 | 1 | 1 | 1 |
|  |  | 50 | 1 | 1 | 1 | 1 | 1 | 1 | 1 | 1 | 1 | 1 | 1 | 0 | 1 | 1 | 1 | 1 | 1 | 1 | 1 | 1 | 1 | 1 |
|  |  | 80 | 2 | 1 | 1 | 1 | 1 | 1 | 1 | 1 | 1 | 1 | 1 | 1 | 1 | 1 | 1 | 1 | 1 | 1 | 1 | 1 | 1 | 1 |
|  | 200 | 10 | 1 | 1 | 1 | 1 | 1 | 1 | 1 | 1 | 1 | 1 | 1 | 1 | 1 | 1 | 1 | 1 | 1 | 1 | 1 | 1 | 1 | 1 |
|  |  | 20 | 2 | 1 | 1 | 1 | 1 | 1 | 1 | 1 | 1 | 1 | 1 | 2 | 1 | 1 | 1 | 1 | 1 | 1 | 1 | 1 | 1 | 1 |
|  |  | 30 | 4 | 1 | 1 | 1 | 1 | 1 | 1 | 1 | 1 | 1 | 1 | 1 | 1 | 1 | 1 | 1 | 1 | 1 | 1 | 1 | 1 | 1 |
|  |  | 50 | 3 | 1 | 1 | 1 | 1 | 1 | 1 | 1 | 1 | 1 | 1 | 2 | 1 | 1 | 1 | 1 | 1 | 1 | 1 | 1 | 1 | 1 |
|  |  | 80 | 1 | 1 | 1 | 1 | 1 | 1 | 1 | 1 | 1 | 1 | 1 | 2 | 1 | 1 | 1 | 1 | 1 | 1 | 1 | 1 | 1 | 1 |
| high | 50 | 10 | 1 | 1 | 1 | 1 | 1 | 1 | 1 | 1 | 1 | 1 | 1 | 0 | 1 | 1 | 1 | 1 | 1 | 1 | 1 | 1 | 1 | 1 |
|  |  | 20 | 0 | 1 | 1 | 1 | 1 | 1 | 1 | 1 | 1 | 1 | 1 | 1 | 1 | 1 | 1 | 1 | 1 | 1 | 1 | 1 | 1 | 1 |
|  |  | 30 | 0 | 1 | 1 | 1 | 1 | 1 | 1 | 1 | 1 | 1 | 1 | 1 | 1 | 1 | 1 | 1 | 1 | 1 | 1 | 1 | 1 | 1 |
|  |  | 50 | 2 | 1 | 1 | 1 | 1 | 1 | 1 | 1 | 1 | 1 | 1 | 1 | 1 | 1 | 1 | 1 | 1 | 1 | 1 | 1 | 1 | 1 |
|  |  | 80 | 0 | 1 | 1 | 1 | 1 | 1 | 1 | 1 | 1 | 1 | 1 | 1 | 1 | 1 | 1 | 1 | 1 | 1 | 1 | 1 | 1 | 1 |
|  | 100 | 10 | 0 | 1 | 1 | 1 | 1 | 1 | 1 | 1 | 1 | 1 | 1 | 0 | 1 | 1 | 1 | 1 | 1 | 1 | 1 | 1 | 1 | 1 |
|  |  | 20 | 1 | 1 | 1 | 1 | 1 | 1 | 1 | 1 | 1 | 1 | 1 | 1 | 1 | 1 | 1 | 1 | 1 | 1 | 1 | 1 | 1 | 1 |
|  |  | 30 | 0 | 1 | 1 | 1 | 1 | 1 | 1 | 1 | 1 | 1 | 1 | 0 | 1 | 1 | 1 | 1 | 1 | 1 | 1 | 1 | 1 | 1 |
|  |  | 50 | 0 | 1 | 1 | 1 | 1 | 1 | 1 | 1 | 1 | 1 | 1 | 0 | 1 | 1 | 1 | 1 | 1 | 1 | 1 | 1 | 1 | 1 |
|  |  | 80 | 3 | 1 | 1 | 1 | 1 | 1 | 1 | 1 | 1 | 1 | 1 | 0 | 1 | 1 | 1 | 1 | 1 | 1 | 1 | 1 | 1 | 1 |
|  | 200 | 10 | 1 | 1 | 1 | 1 | 1 | 1 | 1 | 1 | 1 | 1 | 1 | 0 | 1 | 1 | 1 | 1 | 1 | 1 | 1 | 1 | 1 | 1 |
|  |  | 20 | 0 | 1 | 1 | 1 | 1 | 1 | 1 | 1 | 1 | 1 | 1 | 1 | 1 | 1 | 1 | 1 | 1 | 1 | 1 | 1 | 1 | 1 |
|  |  | 30 | 1 | 1 | 1 | 1 | 1 | 1 | 1 | 1 | 1 | 1 | 1 | 0 | 1 | 1 | 1 | 1 | 1 | 1 | 1 | 1 | 1 | 1 |
|  |  | 50 | 1 | 1 | 1 | 1 | 1 | 1 | 1 | 1 | 1 | 1 | 1 | 0 | 1 | 1 | 1 | 1 | 1 | 1 | 1 | 1 | 1 | 1 |
|  |  | 80 | 1 | 1 | 1 | 1 | 1 | 1 | 1 | 1 | 1 | 1 | 1 | 0 | 1 | 1 | 1 | 1 | 1 | 1 | 1 | 1 | 1 | 1 |

# Results: fit indices’ M and SD for bias condition 2, 3 and 4

Table A6

Fit indices’ means and standard deviations for varying ICCs (low., med. = medium, high), number of participants (n), number of re-tests (t) and for *bias condition 2, 3* and *4* with low/high bias.

|  |  |  |  | Low | | | | | | | | High | | | | | | | |
| --- | --- | --- | --- | --- | --- | --- | --- | --- | --- | --- | --- | --- | --- | --- | --- | --- | --- | --- | --- |
|  |  |  |  | CFI | | RMSEA | | SRMR-b | | SRMR-w | | CFI | | RMSEA | | SRMR-b | | SRMR-w | |
| Bias | ICC | n | t | M | SD | M | SD | M | SD | M | SD | M | SD | M | SD | M | SD | M | SD |
| 2 | low | 50 | 10 | 1 | .002 | .001 | .005 | .021 | .009 | .022 | .005 | .998 | .005 | .005 | .011 | .026 | .013 | .029 | .007 |
|  |  |  | 20 | 1 | .001 | .001 | .005 | .010 | .005 | .016 | .004 | .997 | .005 | .008 | .011 | .013 | .006 | .023 | .005 |
|  |  |  | 30 | 1 | .001 | .001 | .004 | .007 | .003 | .013 | .003 | .996 | .005 | .011 | .011 | .009 | .004 | .022 | .004 |
|  |  |  | 50 | 1 | .001 | .001 | .004 | .004 | .002 | .011 | .003 | .994 | .005 | .016 | .009 | .005 | .003 | .020 | .004 |
|  |  |  | 80 | 1 | .001 | .002 | .004 | .002 | .001 | .009 | .002 | .992 | .004 | .020 | .006 | .003 | .002 | .020 | .003 |
|  |  | 100 | 10 | 1 | .001 | .001 | .004 | .015 | .006 | .016 | .004 | .997 | .004 | .009 | .011 | .019 | .009 | .024 | .005 |
|  |  |  | 20 | 1 | .001 | .001 | .004 | .007 | .003 | .012 | .003 | .995 | .004 | .014 | .010 | .009 | .004 | .021 | .004 |
|  |  |  | 30 | 1 | .001 | .002 | .004 | .005 | .002 | .010 | .002 | .994 | .004 | .018 | .007 | .006 | .003 | .020 | .003 |
|  |  |  | 50 | 1 | .001 | .003 | .004 | .003 | .001 | .009 | .002 | .992 | .003 | .021 | .005 | .004 | .002 | .019 | .003 |
|  |  |  | 80 | .999 | .001 | .004 | .004 | .002 | .001 | .008 | .002 | .990 | .003 | .022 | .003 | .002 | .001 | .019 | .002 |
|  |  | 200 | 10 | 1 | .001 | .001 | .004 | .010 | .004 | .012 | .003 | .996 | .004 | .015 | .010 | .013 | .006 | .021 | .004 |
|  |  |  | 20 | 1 | .001 | .002 | .004 | .005 | .002 | .010 | .002 | .993 | .003 | .020 | .006 | .006 | .003 | .020 | .003 |
|  |  |  | 30 | 1 | .001 | .003 | .004 | .003 | .001 | .008 | .002 | .992 | .003 | .021 | .004 | .004 | .002 | .019 | .002 |
|  |  |  | 50 | .999 | .001 | .004 | .004 | .002 | .001 | .008 | .002 | .991 | .002 | .022 | .003 | .003 | .001 | .019 | .002 |
|  |  |  | 80 | .999 | .001 | .006 | .003 | .001 | 0 | .007 | .001 | .990 | .002 | .023 | .002 | .002 | .001 | .018 | .002 |
|  | med. | 50 | 10 | .999 | .002 | .003 | .009 | .005 | .002 | .026 | .006 | .986 | .012 | .033 | .020 | .007 | .004 | .046 | .009 |
|  |  |  | 20 | .999 | .003 | .006 | .009 | .003 | .001 | .021 | .005 | .976 | .013 | .043 | .014 | .003 | .002 | .043 | .007 |
|  |  |  | 30 | .998 | .003 | .007 | .010 | .002 | .001 | .019 | .004 | .969 | .013 | .046 | .011 | .002 | .001 | .041 | .007 |
|  |  |  | 50 | .997 | .003 | .011 | .009 | .001 | .001 | .017 | .004 | .961 | .013 | .049 | .009 | .001 | .001 | .041 | .006 |
|  |  |  | 80 | .995 | .003 | .015 | .007 | .001 | 0 | .016 | .003 | .955 | .013 | .051 | .008 | .001 | 0 | .041 | .006 |
|  |  | 100 | 10 | .999 | .002 | .006 | .009 | .004 | .002 | .021 | .005 | .981 | .009 | .042 | .012 | .005 | .003 | .043 | .007 |
|  |  |  | 20 | .998 | .003 | .010 | .009 | .002 | .001 | .018 | .004 | .972 | .008 | .048 | .008 | .002 | .001 | .041 | .005 |
|  |  |  | 30 | .997 | .003 | .014 | .008 | .001 | .001 | .017 | .003 | .966 | .008 | .049 | .007 | .002 | .001 | .041 | .005 |
|  |  |  | 50 | .996 | .003 | .017 | .006 | .001 | 0 | .016 | .003 | .959 | .009 | .051 | .006 | .001 | .001 | .041 | .004 |
|  |  |  | 80 | .994 | .002 | .018 | .004 | 0 | 0 | .015 | .002 | .954 | .009 | .051 | .005 | .001 | 0 | .040 | .004 |
|  |  | 200 | 10 | .998 | .002 | .010 | .009 | .003 | .001 | .018 | .003 | .978 | .006 | .047 | .007 | .004 | .002 | .042 | .005 |
|  |  |  | 20 | .997 | .002 | .016 | .006 | .001 | .001 | .016 | .003 | .969 | .006 | .050 | .005 | .002 | .001 | .041 | .004 |
|  |  |  | 30 | .996 | .002 | .018 | .005 | .001 | 0 | .016 | .002 | .964 | .006 | .051 | .005 | .001 | .001 | .041 | .004 |
|  |  |  | 50 | .995 | .002 | .019 | .003 | 0 | 0 | .015 | .002 | .958 | .006 | .052 | .004 | .001 | 0 | .040 | .003 |
|  |  |  | 80 | .994 | .002 | .019 | .003 | 0 | 0 | .015 | .002 | .953 | .006 | .052 | .004 | 0 | 0 | .040 | .003 |
|  | high | 50 | 10 | .996 | .006 | .016 | .018 | .002 | .001 | .035 | .008 | .956 | .018 | .074 | .018 | .003 | .002 | .070 | .011 |
|  |  |  | 20 | .992 | .007 | .025 | .015 | .001 | .001 | .031 | .006 | .930 | .020 | .081 | .013 | .001 | .001 | .068 | .009 |
|  |  |  | 30 | .988 | .008 | .030 | .012 | .001 | 0 | .030 | .006 | .915 | .022 | .084 | .013 | .001 | .001 | .067 | .009 |
|  |  |  | 50 | .983 | .008 | .034 | .009 | 0 | 0 | .029 | .005 | .898 | .026 | .085 | .012 | .001 | 0 | .067 | .009 |
|  |  |  | 80 | .980 | .008 | .035 | .008 | 0 | 0 | .028 | .005 | .883 | .026 | .086 | .011 | 0 | 0 | .066 | .008 |
|  |  | 100 | 10 | .994 | .005 | .024 | .014 | .002 | .001 | .031 | .006 | .950 | .012 | .080 | .011 | .002 | .001 | .068 | .008 |
|  |  |  | 20 | .989 | .005 | .033 | .008 | .001 | 0 | .029 | .005 | .927 | .014 | .084 | .009 | .001 | .001 | .067 | .007 |
|  |  |  | 30 | .985 | .005 | .035 | .007 | .001 | 0 | .028 | .004 | .913 | .015 | .085 | .009 | .001 | 0 | .067 | .006 |
|  |  |  | 50 | .982 | .005 | .036 | .006 | 0 | 0 | .028 | .004 | .895 | .018 | .087 | .009 | 0 | 0 | .067 | .006 |
|  |  |  | 80 | .979 | .006 | .037 | .005 | 0 | 0 | .027 | .004 | .881 | .019 | .087 | .008 | 0 | 0 | .066 | .006 |
|  |  | 200 | 10 | .992 | .004 | .032 | .008 | .001 | .001 | .029 | .004 | .947 | .009 | .082 | .008 | .002 | .001 | .067 | .006 |
|  |  |  | 20 | .987 | .004 | .035 | .005 | .001 | 0 | .028 | .003 | .925 | .010 | .085 | .007 | .001 | 0 | .067 | .005 |
|  |  |  | 30 | .985 | .004 | .036 | .004 | 0 | 0 | .028 | .003 | .910 | .011 | .087 | .006 | 0 | 0 | .067 | .005 |
|  |  |  | 50 | .981 | .004 | .037 | .004 | 0 | 0 | .027 | .003 | .894 | .013 | .087 | .006 | 0 | 0 | .067 | .005 |
|  |  |  | 80 | .978 | .004 | .038 | .004 | 0 | 0 | .027 | .002 | .879 | .014 | .088 | .006 | 0 | 0 | .067 | .004 |
| 3 | low | 50 | 10 | 1 | .002 | .001 | .005 | .021 | .010 | .022 | .005 | .998 | .005 | .004 | .010 | .026 | .013 | .028 | .007 |
|  |  |  | 20 | 1 | .001 | .001 | .004 | .010 | .004 | .016 | .004 | .997 | .005 | .008 | .011 | .013 | .007 | .024 | .005 |
|  |  |  | 30 | 1 | .001 | .001 | .004 | .007 | .003 | .013 | .003 | .996 | .005 | .011 | .010 | .008 | .004 | .022 | .004 |
|  |  |  | 50 | 1 | .001 | .001 | .004 | .004 | .002 | .011 | .003 | .994 | .005 | .016 | .008 | .005 | .002 | .021 | .004 |
|  |  |  | 80 | 1 | .001 | .002 | .004 | .002 | .001 | .009 | .002 | .992 | .004 | .019 | .006 | .003 | .002 | .020 | .003 |
|  |  | 100 | 10 | 1 | .001 | .001 | .005 | .014 | .006 | .016 | .004 | .997 | .004 | .009 | .011 | .018 | .008 | .024 | .005 |
|  |  |  | 20 | 1 | .001 | .002 | .004 | .007 | .003 | .012 | .003 | .995 | .004 | .015 | .010 | .009 | .004 | .021 | .004 |
|  |  |  | 30 | 1 | .001 | .002 | .004 | .005 | .002 | .010 | .002 | .994 | .004 | .018 | .007 | .006 | .003 | .020 | .003 |
|  |  |  | 50 | 1 | .001 | .002 | .004 | .003 | .001 | .009 | .002 | .992 | .004 | .021 | .005 | .004 | .002 | .020 | .003 |
|  |  |  | 80 | 1 | .001 | .003 | .004 | .002 | .001 | .008 | .002 | .990 | .003 | .022 | .004 | .002 | .001 | .019 | .002 |
|  |  | 200 | 10 | 1 | .001 | .002 | .004 | .010 | .004 | .012 | .003 | .996 | .004 | .014 | .010 | .013 | .005 | .021 | .004 |
|  |  |  | 20 | 1 | .001 | .002 | .004 | .005 | .002 | .010 | .002 | .993 | .003 | .020 | .005 | .006 | .003 | .020 | .003 |
|  |  |  | 30 | 1 | .001 | .003 | .004 | .003 | .001 | .008 | .002 | .992 | .003 | .021 | .004 | .004 | .002 | .019 | .002 |
|  |  |  | 50 | .999 | .001 | .004 | .004 | .002 | .001 | .008 | .001 | .991 | .002 | .022 | .003 | .002 | .001 | .019 | .002 |
|  |  |  | 80 | .999 | .001 | .006 | .003 | .001 | 0 | .007 | .001 | .989 | .002 | .023 | .002 | .002 | .001 | .019 | .002 |
|  | med. | 50 | 10 | .999 | .002 | .003 | .008 | .005 | .003 | .026 | .006 | .988 | .011 | .028 | .020 | .006 | .003 | .047 | .009 |
|  |  |  | 20 | .999 | .003 | .005 | .009 | .003 | .001 | .021 | .005 | .976 | .012 | .042 | .013 | .003 | .002 | .044 | .007 |
|  |  |  | 30 | .998 | .003 | .008 | .010 | .002 | .001 | .019 | .004 | .969 | .013 | .046 | .010 | .002 | .001 | .043 | .007 |
|  |  |  | 50 | .997 | .003 | .011 | .009 | .001 | 0 | .017 | .004 | .961 | .012 | .048 | .008 | .001 | .001 | .042 | .006 |
|  |  |  | 80 | .996 | .003 | .015 | .007 | .001 | 0 | .016 | .003 | .954 | .013 | .050 | .008 | .001 | 0 | .042 | .006 |
|  |  | 100 | 10 | .999 | .002 | .005 | .009 | .004 | .002 | .021 | .004 | .982 | .009 | .041 | .013 | .004 | .002 | .044 | .007 |
|  |  |  | 20 | .998 | .002 | .010 | .009 | .002 | .001 | .018 | .004 | .971 | .009 | .047 | .008 | .002 | .001 | .043 | .006 |
|  |  |  | 30 | .997 | .003 | .013 | .008 | .001 | .001 | .017 | .003 | .965 | .009 | .049 | .007 | .001 | .001 | .042 | .005 |
|  |  |  | 50 | .995 | .003 | .016 | .006 | .001 | 0 | .016 | .003 | .958 | .009 | .050 | .006 | .001 | 0 | .042 | .005 |
|  |  |  | 80 | .994 | .003 | .018 | .004 | 0 | 0 | .016 | .002 | .952 | .010 | .051 | .006 | .001 | 0 | .042 | .004 |
|  |  | 200 | 10 | .999 | .002 | .009 | .009 | .002 | .001 | .018 | .004 | .979 | .006 | .046 | .007 | .003 | .001 | .043 | .005 |
|  |  |  | 20 | .997 | .002 | .015 | .007 | .001 | .001 | .017 | .003 | .970 | .006 | .049 | .005 | .001 | .001 | .042 | .004 |
|  |  |  | 30 | .996 | .002 | .017 | .005 | .001 | 0 | .016 | .002 | .964 | .006 | .050 | .005 | .001 | .001 | .042 | .004 |
|  |  |  | 50 | .995 | .002 | .019 | .003 | 0 | 0 | .016 | .002 | .957 | .006 | .051 | .004 | .001 | 0 | .042 | .003 |
|  |  |  | 80 | .994 | .002 | .019 | .003 | 0 | 0 | .015 | .002 | .952 | .007 | .051 | .004 | 0 | 0 | .041 | .003 |
|  | high | 50 | 10 | .997 | .005 | .014 | .017 | .002 | .001 | .036 | .007 | .959 | .018 | .070 | .018 | .003 | .002 | .073 | .011 |
|  |  |  | 20 | .992 | .007 | .023 | .015 | .001 | .001 | .032 | .006 | .932 | .020 | .079 | .013 | .001 | .001 | .071 | .010 |
|  |  |  | 30 | .988 | .007 | .029 | .012 | .001 | 0 | .031 | .006 | .915 | .023 | .082 | .013 | .001 | .001 | .071 | .010 |
|  |  |  | 50 | .983 | .008 | .033 | .009 | 0 | 0 | .030 | .005 | .896 | .025 | .084 | .011 | .001 | 0 | .070 | .009 |
|  |  |  | 80 | .980 | .008 | .035 | .008 | 0 | 0 | .029 | .005 | .878 | .028 | .085 | .011 | 0 | 0 | .070 | .009 |
|  |  | 100 | 10 | .995 | .005 | .022 | .015 | .002 | .001 | .032 | .006 | .953 | .012 | .076 | .011 | .002 | .001 | .071 | .008 |
|  |  |  | 20 | .989 | .005 | .031 | .009 | .001 | 0 | .030 | .005 | .928 | .014 | .082 | .009 | .001 | 0 | .071 | .007 |
|  |  |  | 30 | .986 | .005 | .033 | .007 | 0 | 0 | .029 | .004 | .912 | .016 | .084 | .009 | .001 | 0 | .070 | .007 |
|  |  |  | 50 | .982 | .005 | .035 | .006 | 0 | 0 | .029 | .004 | .893 | .018 | .085 | .008 | 0 | 0 | .070 | .007 |
|  |  |  | 80 | .978 | .006 | .036 | .005 | 0 | 0 | .029 | .004 | .878 | .020 | .085 | .008 | 0 | 0 | .070 | .006 |
|  |  | 200 | 10 | .993 | .004 | .030 | .009 | .001 | 0 | .030 | .004 | .950 | .009 | .079 | .008 | .001 | .001 | .071 | .006 |
|  |  |  | 20 | .988 | .004 | .034 | .005 | .001 | 0 | .029 | .003 | .925 | .010 | .084 | .006 | .001 | 0 | .071 | .005 |
|  |  |  | 30 | .984 | .004 | .036 | .004 | 0 | 0 | .029 | .003 | .911 | .011 | .084 | .006 | 0 | 0 | .070 | .005 |
|  |  |  | 50 | .981 | .004 | .037 | .004 | 0 | 0 | .029 | .003 | .893 | .012 | .085 | .006 | 0 | 0 | .070 | .004 |
|  |  |  | 80 | .977 | .004 | .037 | .003 | 0 | 0 | .028 | .003 | .877 | .014 | .086 | .006 | 0 | 0 | .070 | .004 |
| 4 | low | 50 | 10 | .998 | .005 | .005 | .010 | .024 | .011 | .028 | .007 | .962 | .026 | .041 | .019 | .034 | .016 | .054 | .010 |
|  |  |  | 20 | .997 | .005 | .007 | .010 | .012 | .006 | .023 | .005 | .944 | .021 | .050 | .011 | .017 | .009 | .051 | .008 |
|  |  |  | 30 | .996 | .005 | .010 | .010 | .008 | .004 | .021 | .004 | .934 | .020 | .053 | .009 | .012 | .006 | .050 | .007 |
|  |  |  | 50 | .994 | .005 | .015 | .009 | .005 | .002 | .020 | .004 | .923 | .018 | .055 | .007 | .007 | .003 | .049 | .006 |
|  |  |  | 80 | .992 | .005 | .018 | .006 | .003 | .001 | .019 | .003 | .914 | .018 | .056 | .006 | .004 | .002 | .049 | .006 |
|  |  | 100 | 10 | .997 | .004 | .008 | .011 | .017 | .007 | .023 | .005 | .951 | .018 | .050 | .010 | .025 | .011 | .051 | .007 |
|  |  |  | 20 | .996 | .004 | .013 | .010 | .008 | .003 | .020 | .004 | .937 | .014 | .054 | .007 | .012 | .005 | .050 | .006 |
|  |  |  | 30 | .994 | .004 | .017 | .008 | .006 | .002 | .019 | .003 | .929 | .013 | .056 | .006 | .008 | .004 | .049 | .005 |
|  |  |  | 50 | .992 | .004 | .020 | .005 | .003 | .001 | .018 | .003 | .920 | .012 | .057 | .005 | .005 | .002 | .049 | .004 |
|  |  |  | 80 | .990 | .003 | .021 | .004 | .002 | .001 | .018 | .002 | .912 | .013 | .057 | .005 | .003 | .001 | .049 | .004 |
|  |  | 200 | 10 | .996 | .004 | .014 | .010 | .012 | .005 | .021 | .004 | .945 | .012 | .054 | .006 | .018 | .008 | .050 | .005 |
|  |  |  | 20 | .994 | .003 | .019 | .006 | .006 | .002 | .019 | .003 | .934 | .010 | .056 | .005 | .009 | .004 | .049 | .004 |
|  |  |  | 30 | .992 | .003 | .020 | .004 | .004 | .001 | .018 | .002 | .927 | .009 | .057 | .004 | .006 | .002 | .049 | .004 |
|  |  |  | 50 | .991 | .002 | .021 | .003 | .002 | .001 | .018 | .002 | .918 | .009 | .057 | .004 | .003 | .001 | .049 | .003 |
|  |  |  | 80 | .989 | .002 | .022 | .002 | .001 | .001 | .017 | .002 | .911 | .008 | .057 | .003 | .002 | .001 | .048 | .003 |
|  | med. | 50 | 10 | .985 | .013 | .032 | .021 | .006 | .003 | .047 | .009 | .870 | .032 | .107 | .017 | .010 | .005 | .110 | .016 |
|  |  |  | 20 | .973 | .014 | .044 | .013 | .003 | .001 | .044 | .008 | .819 | .036 | .113 | .015 | .005 | .003 | .108 | .015 |
|  |  |  | 30 | .966 | .015 | .046 | .011 | .002 | .001 | .042 | .008 | .788 | .038 | .114 | .014 | .003 | .002 | .107 | .014 |
|  |  |  | 50 | .958 | .014 | .048 | .009 | .001 | .001 | .041 | .007 | .750 | .043 | .116 | .014 | .002 | .001 | .106 | .014 |
|  |  |  | 80 | .950 | .016 | .050 | .008 | .001 | 0 | .041 | .007 | .717 | .048 | .116 | .014 | .001 | .001 | .106 | .014 |
|  |  | 100 | 10 | .980 | .010 | .043 | .012 | .005 | .002 | .044 | .007 | .860 | .022 | .111 | .011 | .007 | .004 | .108 | .012 |
|  |  |  | 20 | .969 | .010 | .048 | .008 | .002 | .001 | .042 | .006 | .812 | .026 | .115 | .011 | .003 | .002 | .107 | .011 |
|  |  |  | 30 | .962 | .010 | .050 | .007 | .001 | .001 | .042 | .005 | .782 | .028 | .116 | .010 | .002 | .001 | .107 | .010 |
|  |  |  | 50 | .955 | .011 | .051 | .006 | .001 | 0 | .041 | .005 | .746 | .032 | .117 | .010 | .001 | .001 | .106 | .010 |
|  |  |  | 80 | .948 | .011 | .051 | .006 | .001 | 0 | .041 | .005 | .715 | .035 | .117 | .010 | .001 | 0 | .106 | .010 |
|  |  | 200 | 10 | .977 | .007 | .048 | .007 | .003 | .001 | .042 | .005 | .854 | .017 | .114 | .009 | .005 | .003 | .108 | .009 |
|  |  |  | 20 | .967 | .007 | .050 | .005 | .002 | .001 | .041 | .004 | .809 | .018 | .116 | .008 | .003 | .001 | .107 | .008 |
|  |  |  | 30 | .961 | .007 | .051 | .005 | .001 | 0 | .041 | .004 | .781 | .020 | .117 | .007 | .002 | .001 | .106 | .007 |
|  |  |  | 50 | .953 | .007 | .052 | .004 | .001 | 0 | .041 | .004 | .743 | .022 | .118 | .007 | .001 | .001 | .107 | .007 |
|  |  |  | 80 | .947 | .008 | .052 | .004 | 0 | 0 | .041 | .003 | .712 | .026 | .118 | .007 | .001 | 0 | .107 | .007 |
|  | high | 50 | 10 | .946 | .023 | .079 | .020 | .003 | .002 | .079 | .015 | .835 | .031 | .143 | .015 | .005 | .015 | .147 | .014 |
|  |  |  | 20 | .916 | .026 | .085 | .015 | .001 | .001 | .077 | .013 | .620 | .035 | .160 | .006 | 0 | 0 | .150 | .006 |
|  |  |  | 30 | .899 | .029 | .087 | .015 | .001 | 0 | .076 | .013 | .725 | .026 | .153 | .009 | .001 | .001 | .147 | .009 |
|  |  |  | 50 | .874 | .034 | .089 | .014 | .001 | 0 | .076 | .012 | .673 | .029 | .156 | .008 | .002 | .021 | .147 | .008 |
|  |  |  | 80 | .854 | .038 | .090 | .013 | 0 | 0 | .075 | .012 | .630 | .036 | .158 | .008 | .001 | .016 | .148 | .008 |
|  |  | 100 | 10 | .940 | .015 | .084 | .012 | .002 | .001 | .077 | .010 | .825 | .022 | .148 | .010 | .003 | .002 | .148 | .010 |
|  |  |  | 20 | .913 | .017 | .088 | .010 | .001 | 0 | .076 | .009 | .757 | .018 | .154 | .007 | .001 | .001 | .148 | .006 |
|  |  |  | 30 | .893 | .021 | .089 | .010 | .001 | 0 | .076 | .009 | .716 | .023 | .156 | .006 | .001 | 0 | .149 | .006 |
|  |  |  | 50 | .871 | .024 | .090 | .009 | 0 | 0 | .075 | .009 | .667 | .025 | .158 | .005 | .001 | .015 | .149 | .005 |
|  |  |  | 80 | .852 | .028 | .090 | .010 | 0 | 0 | .075 | .009 | .624 | .033 | .159 | .006 | .001 | .015 | .149 | .006 |
|  |  | 200 | 10 | .937 | .011 | .087 | .008 | .002 | .001 | .077 | .007 | .716 | .023 | .156 | .006 | .001 | .001 | .149 | .006 |
|  |  |  | 20 | .909 | .013 | .090 | .007 | .001 | 0 | .076 | .007 | .753 | .017 | .155 | .005 | .001 | .001 | .149 | .005 |
|  |  |  | 30 | .892 | .015 | .090 | .007 | .001 | 0 | .076 | .006 | .712 | .022 | .157 | .006 | .001 | .015 | .149 | .005 |
|  |  |  | 50 | .869 | .016 | .091 | .007 | 0 | 0 | .076 | .006 | .662 | .025 | .159 | .005 | 0 | 0 | .150 | .005 |
|  |  |  | 80 | .851 | .020 | .091 | .007 | 0 | 0 | .075 | .006 | .619 | .033 | .160 | .005 | 0 | 0 | .151 | .006 |
